# A 3D ovarian cancer metastasis model using a decellularised peritoneal matrix to study therapy response

**DOI:** 10.1016/j.ebiom.2026.106135

**Published:** 2026-02-02

**Authors:** Christiane Helgestad Gjerde, Katrin Kleinmanns, Anika Langer, Gorka Ruiz de Garibay Ponce, Ezekiel Rozmus, Gina Nyhus Stangeland, Calum Leitch, Rammah Elnour, Harsh Nitin Dongre, Constantin Berger, Okan Gultekin, Christopher Forcados, Maria Stensland, Tuula Anneli Nyman, Kaisa Lehti, Ben Davidson, Sébastien Wälchli, Pascal Gelebart, Daniela Elena Costea, Spiros Kotopoulis, Line Bjørge, Emmet McCormack

**Affiliations:** a Centre for Cancer Biomarkers CCBIO, Department of Clinical Science, University of Bergen, Jonas Lies vei 87, 5021, Bergen, Norway; b Department of Obstetrics and Gynecology, Haukeland University Hospital, Haukelandsbakken 15, 5021, Bergen, Norway; c Department of Physiology, University of Kentucky, 741 S. Limestone, BBSRB, B243, Lexington, KY, 40536, USA; d Gade Laboratory of Pathology, Department of Clinical Medicine, University of Bergen, Jonas Lies vei 91 B, 5021, Bergen, Norway; e Department for Functional Materials in Medicine and Dentistry, University Hospital Würzburg, Röntgenring 11, Würzburg, Germany; f Department of Microbiology, Tumour and Cell Biology, Karolinska Institutet, Solnavägen 9, 17165, Solna, Stockholm, Sweden; g Faculty of Medicine, University of Oslo, Sognsvannsveien 9, 0372, Oslo, Norway; h Department of Immunology, University of Oslo and Oslo University Hospital, Sognsvannsveien 20, Rikshospitalet, 0372, Oslo, Norway; i Department of Biomedical Laboratory Science, Norwegian University of Science and Technology, Erling Skjalgssons Gate 1, 7030, Trondheim, Norway; j Department of Pathology, Oslo University Hospital, Norwegian Radium Hospital, Ullernchausséen 70, 0379, Oslo, Norway; k Faculty of Medicine, Institute of Clinical Medicine, University of Oslo, Kirkeveien 166, 0450, Oslo, Norway; l Translational Research Unit, Department of Cellular Therapy, Oslo University Hospital, Ullernchausséen 64, 0379, Oslo, Norway; m Centre for Cancer Biomarkers CCBIO and Gade Laboratory of Pathology, Department of Clinical Medicine, University of Bergen, Jonas Lies vei 91 B, 5021, Bergen, Norway; n Department of Pathology, Haukeland University Hospital, Jonas Lies vei 91 B, 5021, Bergen, Norway; o National Centre for Ultrasound in Gastroenterology, Haukeland University Hospital, Jonas Lies vei 83, 5021, Bergen, Norway; p Department of Clinical Medicine, University of Bergen, Jonas Lies vei 91 B, 5021, Bergen, Norway; q Centre for Pharmacy, Department of Clinical Science, University of Bergen, Jonas Lies vei 87, 5021, Bergen, Norway; r K.G. Jebsen Center for Myeloid Blood Cancer, Department of Clinical Science, University of Bergen, Jonas Lies vei 87, 5021, Bergen, Norway; s Department of Hematology, Haukeland University Hospital, Jonas Lies vei 83, 5021, Bergen, Norway

**Keywords:** HGSOC, Peritoneal metastases, 3D model, dECM scaffold, Proteomics, Drug sensitivity, CAR T

## Abstract

**Background:**

High-grade serous ovarian carcinoma (HGSOC) presents a significant therapeutic challenge. Late-stage disease is frequently associated with peritoneal carcinomatosis. The peritoneal metastases exhibit a unique tumour microenvironment (TME) distinct from the primary tumours and other metastatic sites. Understanding the critical influence of the extracellular matrix (ECM) in shaping the tumour phenotype is essential for the development of effective new therapies.

**Methods:**

This study introduces a three-dimensional (3D) model of HGSOC peritoneal metastases using a porcine decellularised peritoneal-derived ECM scaffold, referred to as peritoneal matrix (PerMa).

**Findings:**

We show that the decellularisation maintains the structural integrity and composition of ECM molecules. Comparative analysis reveals structural, compositional, and mechanical similarities between porcine and human peritoneal matrices, underscoring the porcine model's translational relevance for modelling human peritoneum physiology. The PerMa supports the 3D growth of HGSOC cell lines. The model enables the assessment of sensitivity to traditional chemotherapy and novel cell-based immunotherapy through confocal imaging and quantification of cell volume.

**Interpretation:**

Our model offers a valuable platform for investigating peritoneal carcinomatosis in HGSOC, with the potential to contribute significantly to developing novel therapeutic approaches.

**Funding:**

Financial support was provided by the 10.13039/501100005036University of Bergen, Helse Vest RHF (F-12183-D10616, 779, 911182, 912035, and 912146), Helse Bergen HF (240222), the 10.13039/100008730Norwegian Cancer Society (6833652 and 182735), the 10.13039/501100005416Research Council of Norway grants (250317, 326300, 223250, 262652, and 295910), the Novo Nordisk Foundation (NNF21OC0070381), the Kolbjørn Brambani Legat for Kreftforskning, the National Institute of Health (R01CA199646) and the 10.13039/501100002794Swedish Cancer Society (21 1888 Pj).

**Research in context**

**Evidence before this study**

High-grade serous ovarian carcinoma (HGSOC) represents a significant therapeutic challenge due to widespread peritoneal metastases. Current evidence highlights the importance of the tumour microenvironment (TME), particularly the extracellular matrix (ECM), in modulating tumour behaviour in HGSOC. Current preclinical models often fail to recapitulate the complexity of the human peritoneal ECM, limiting their predictive value for therapeutic interventions.

**Added value of this study**

In this study, we have developed a 3D model of HGSOC peritoneal metastases utilising cancer cells alone or in coculture with fibroblasts on a dECM scaffold derived from porcine peritoneum, referred to as PerMa. Unlike previous models, the PerMa recapitulates the microenvironment of the peritoneal ECM, providing a physiologically relevant microenvironment to study the biology of HGSOC peritoneal metastases and discover new therapeutic targets.

**Implications of all the available evidence**

Our study demonstrates the utility of a 3D model of HGSOC with a relevant ECM compartment. The PerMa facilitates investigations into tumour-ECM interactions, which are essential for understanding HGSOC progression. The model offers a valuable preclinical tool for testing novel therapeutic targets, including cell-based immunotherapies, with potential applications in personalised medicine. Additionally, the model could be used to study other diseases of the peritoneum, including metastases from other malignancies and endometriosis. Ultimately, this work contributes to the development of targeted therapeutic approaches aimed at improving patient outcomes in HGSOC with peritoneal metastases.

## Introduction

Epithelial ovarian cancer (EOC) is the most lethal gynaecological malignancy with an estimated 310,000 new cases and 200,000 new deaths every year.^1^ The prognosis for patients heavily depends on the stage at diagnosis, with most being diagnosed at a late stage, resulting in a 5-year survival rate of less than 30% for this group.^2^^,^^3^ High-grade serous ovarian carcinoma (HGSOC) is the most aggressive subtype of EOC, accounting for the majority of EOC-related deaths.^4^^,^^5^ HGSOC primarily spreads across the peritoneal cavity, referred to as transcoelomic metastasis, forming metastatic deposits on the mesothelial lining of the cavity.^6^^,^^7^ The peritoneum, a specialised serous membrane, lines the abdominal walls (peritoneum parietale) and internal organs (peritoneum viscerale).^8^ The malignant cells follow the dynamics of the peritoneal fluid and preferentially distribute to areas with constant exposure to peritoneal fluid (the omentum) and areas where fluid accumulates (the Pouch of Douglas and the right subphrenic region). This pattern of spread leads to peritoneal carcinomatosis, marked by widespread and diffuse metastases that significantly complicate surgical resection.^9^

Metastases on the visceral and parietal peritoneum are typically numerous, small, and superficial, with distinct phenotypic features compared to omental metastases and other metastatic sites.^10^, ^11^, ^12^, ^13^ Examining HGSOC transcriptome and histology, Pietilä et al. describe the fibrotic ECM heterogeneity at ovarian tumours and other metastatic sites, including the peritoneum and the omentum in treatment naïve and treated HGSOCs. These results also demonstrate that the desmoplastic ECM signalling enhances HGSOC chemoresistance by ECM composition- and stiffness-induced β1integrin-FAK-pMLC-YAP signalling.^13^ A recent study by Vázquez-García et al. underscored how differences in the local microenvironment at various metastatic sites influence the formation of the tumour microenvironment (TME).^11^ They observed disparities in tumour-immune cell interactions between HGSOC ovarian tumours and metastases from different anatomical sites, such as bowel and adipose tissue. How the different cellular and non-cellular components of the local TME impact these interactions remains to be elucidated.

The extracellular matrix (ECM) plays an important role in disease aggressiveness and therapy responses.^14^^,^^15^ The ECM is a macromolecular network of proteins and polysaccharides that provides physical scaffolding for cells and regulates cell differentiation, proliferation, migration, and drug resistance.^16^ In the metastatic process, HGSOC cells adhere to the peritoneal lining and invade the underlying stroma.^17^ They contribute to ECM remodelling through interaction with peritoneal cells, which promotes metastasis.^18^ Further, ovarian cancer–associated mesothelial cells within the peritoneum can induce acquired platinum resistance via fibronectin (FN1)/Akt signalling, highlighting the active role of ECM–cell interactions in modulating chemotherapy response.^19^ ECM remodelling during tumour progression and in response to chemotherapy also affects immune cell infiltration, which in turn can influence the response to immunotherapeutics.^20^, ^21^, ^22^

Given that it is the metastatic disease that ultimately leads to high patient mortality in HGSOC, improving our understanding of adhesion, invasion, and ECM remodelling in HGSOC metastases is crucial. To achieve this, we need advanced models that accurately replicate the complexity of the different metastatic sites, for better disease modelling and drug testing. Three-dimensional (3D) *in vitro* models recapitulate different aspects of tumour biology. Patient-derived organoids (PDOs) have been shown to preserve the genomic features of the original patient tumour.^23^, ^24^, ^25^, ^26^, ^27^ However, 3D cultures based on patient-derived cancer stem cells fail to model the cell–cell and cell–matrix interactions within the TME.^28^ On the other hand, multicellular 3D organotypic models more accurately mimic the heterogeneous *in vivo* tissue architecture.^28^ Despite this improvement, most models still rely on hydrogels, such as collagen I gels or Matrigel®, which inadequately replicate the complex ECM of tissues. The patient-derived omental matrix gel (OmGel) mimics the HGSOC omental metastasis protein composition and is shown to be a promising tool for studying the biology of omental metastases.^29^ However, the complex architecture of the ECM is not well preserved in hydrogels. Biological matrices generated by tissue decellularisation offer a promising alternative with a preserved ECM structure and biomolecules critical for cell behaviour and response to therapy.^30^ Small intestinal submucosa (SIS) derived from pigs has been used to generate 3D models of colorectal cancer, and more recently EOC.^31^^,^^32^ Similarly, Puttock et al. generated a dECM scaffold from human omental metastases and demonstrated that normal macrophages cultured on the scaffold acquired a transcriptional profile similar to tumour-associated macrophages (TAMs).^33^ In a related approach, Sensi et al. developed a patient-derived 3D model using dECM from metastatic HGSOC tumour tissue, demonstrating that the tumour-specific ECM can support cancer cell growth.^34^ Considering the heterogeneity in the interconnected ECM and immune TME across metastatic sites, experimental models that accurately mimic the specific ECM environments are of critical need to study and target the mechanisms of HGSOC metastasis and therapy response and resistance.^11^^,^^13^ Thus, a peritoneal decellularised ECM (dECM) scaffold would be preferred to model peritoneal carcinomatosis.

Although most established 3D models of HGSOC metastases have primarily focused on the omentum, in this study, we developed a dECM scaffold referred to as a peritoneal matrix (PerMa) from porcine and human parietal peritoneum to better mimic the peritoneal ECM. The decellularisation effectively removed cells and genetic material while maintaining the structural integrity of major ECM components. Due to the observed resemblance to the human peritoneum in this study, the porcine PerMa (pPerMa) was used, offering advantages as a scaffold for 3D culture in terms of availability and reproducibility. The peritoneal scaffold supported the 3D growth of HGSOC cell lines. We further demonstrated the utility of the model for assessing sensitivity to traditional chemotherapy and novel, cell-based immunotherapy. The 3D PerMa model is a promising platform for studying the complex interplay between HGSOC cells and the peritoneal microenvironment and how the interaction affects therapy sensitivity.

## Methods

### Materials

#### Cell lines and reagents

Human EOC cell lines Caov3 (cat# HTB-75, RRID: CVCL_0201), OV90 (cat# CRL-11732, RRID: CVCL_3768), and OVCAR3 (cat # HTB-161, RRID: CVCL_0465) were obtained from the American Type Culture Collection (ATCC Manassas, VA, USA), and human EOC cell line COV318 (cat# 07071903, RRID: CVCL_2419) from Sigma Aldrich (Sigma Aldrich, St. Louis, MO, USA). The human fibroblast cell line BJ was kindly provided by Professor Donald Gullberg (Department of Biomedicine, University of Bergen, Norway; cat# CRL-2522, RRID: CVCL-3653). BJ cells are fibroblasts established from skin taken from normal foreskin from a neonatal male. Cell lines were validated by STR profiling and tested for Mycoplasma contamination (Supplementary Files).

The cell lines were cultured in RPMI 1640 (OV-90) or DMEM high glucose (Caov-3, COV318, BJ fibroblasts) media supplemented with 10% heat-inactivated foetal bovine serum (FBS), 1% l-glutamine and 1% penicillin-streptomycin (P/S) (RPMI cat# R5886, DMEM cat# D5671, penicillin-streptomycin cat# P0781, Sigma Aldrich; FBS cat# 10270106, l-Glutamine cat# 25030081, Gibco, Waltham, MA, USA). For co-culture experiments with OV90 and BJ fibroblasts, the cells were cultured in a 1:1 ratio of RPMI/DMEM.

Human EOC cell lines Caov-3, OV-90, COV318, and OVCAR3 were transduced to express green fluorescent protein (GFP), performed according to the manufacturer's protocol using the Redi-Fect Red-FLuc-GFP lentiviral particles (cat# CLS960003, Perkin Elmer, Waltham, MA, USA). The BJ fibroblast cell line was transduced to express miRFP670, performed according to the manufacturer's protocol using the pLenti6.2_miRFP670 lentiviral particles (pLenti6.2_miRFP670 was a gift from Vanessa LaPointe, Addgene plasmid# 113726; http://n2t.net/addgene:113726; RRID: Addgene_113726). PBMCs were obtained from healthy donors at Oslo University Hospital.

#### Human and animal tissues

Porcine small intestine and peritoneum were surplus material from healthy adult domestic pigs (*Sus scrofa*) used for surgical training or in research at the Laboratory Animal Facility, Department of Clinical Medicine, University of Bergen, Norway. The samples were collected shortly post-mortem, within 1 h. Both male and female animals were used. No live animal procedures were performed specifically for this study. Tissue was collected from n = 20 pigs.

Human healthy peritoneum was obtained from autopsies of women at the Department of Pathology, Haukeland University Hospital, Bergen, Norway. Women with intra-abdominal inflammation, cancer, or peritoneal carcinomatosis were excluded from the study. The material was collected within 48 h after death.

Peritoneum from patients with ovarian cancer was collected from chemotherapy naïve patients with primary advanced high-grade serous ovarian carcinoma (HGSOC), admitted to the Department of Obstetrics and Gynecology, Haukeland University Hospital, Bergen, Norway. The peritoneum was collected from a region with no macroscopically visible metastasis. The peritoneal tissue specimens were included in the Bergen Gynaecologic Cancer Biobank.

Due to the limited availability of tissue from both HGSOC patients and autopsies, it was not possible to match healthy individuals directly to patients in this study. However, the age and gender distribution of the autopsy cohort was comparable to that of the HGSOC cohort, supporting their use as a healthy reference group.

### Ethics

Animal experiments were performed in accordance with the procedures from the Norwegian Commission for Laboratory Animals, the European Convention for the Protection of Vertebrates Used for Scientific Purposes (EU Directive 2010/63/EU for animal experiments), and the ethical standards given in the Declaration of Helsinki of 2008.

The surgical training and the research experiments in animals were approved by the Norwegian Food Safety Authority (FOTS IDs: 29559, 13911, 13904, 10294, 20330, and 21065). According to The Norwegian State Commission for Laboratory Animals, there was no need for additional approvals from the Norwegian Food Safety Authority for the use of remnant material from the dead test animals.

The regional committee for Medical and Health Research Ethics (REK West or REK South East) approved the biobank and the studies (Reference IDs: 2014/1907, 30477, 2019/121).

For the collection of human healthy peritoneum from autopsies, informed consent was obtained from the patient's closest relative before the collection of material. For the collection of peritoneum from patients with ovarian cancer, written informed consent was obtained from the women before collection of the samples.

### Generation of SIS from porcine intestines

Production of small intestinal submucosa matrix (SIS) by decellularisation of porcine jejunum was performed as described previously.^35^ Briefly, segments of jejunum were cleaned with tap water and cut into 20 cm segments. Intestinal segments were inverted, and mucosa was scraped off using forceps, followed by incubation overnight in Ca^2+^- and Mg^2+^-free phosphate buffered saline (PBS—) with 1% P/S. Prior to decellularisation, specimens for DNA quantification, proteomic and histopathological analysis were collected. Samples for histopathological analysis were fixed in 4% paraformaldehyde (PFA) for 1 h at room temperature and paraffin-embedded. Samples for DNA quantification and proteomics were frozen at −80 °C. For tissue decellularisation, intestinal segments were first filled with 3.4% sodium deoxycholate (DOC; cat #D6750, Sigma Aldrich, St. Louis, USA) dissolved in Milli-Q (MQ) water and incubated at 4 °C for 1.5 h with a magnetic stirrer. After washing 5 times during 1 h with PBS^—^ containing 1% P/S, SIS pieces were stored in PBS^—^ containing 1% P/S overnight. The following day, SIS pieces were incubated with 0.167 mg/mL DNAse I (cat # 10104159001, Roche, Basel, Switzerland) in PBS with Ca^2+^ and Mg^2+^ (PBS^+^; cat # D8662, Sigma Aldrich, St. Louis, USA) for 2 h at 37 °C under continuous agitation, then washed 3 times during 1 h with PBS^—^. Samples for histopathological analysis, DNA quantification, and proteomics were collected as described above. SIS pieces were stored in PBS^—^ containing 1% P/S at 4 °C. Generated SIS scaffolds were g-sterilised with a dosage of 25 kGy (Gammatom srl, Guanzate, Italy) and stored in PBS^—^ with 1% P/S at 4 °C until further use.

### Generation of PerMa from porcine and human peritoneum

Peritoneal matrix (PerMa) was generated by decellularisation of parietal peritoneum from the anterior abdominal walls from three different sources: pigs, autopsy of women with healthy peritoneum, and women with ovarian cancer undergoing primary cytoreductive surgery. Before the decellularisation, samples for DNA quantification, proteomic analysis, and histopathological analysis were collected, as described for SIS. After sampling, the peritoneum was washed for a minimum of 1 h and up to 18 h with PBS^—^ with 1% P/S. Submesothelial fat was removed by blunt dissection to decrease the thickness of the membranes and improve decellularisation efficiency. For decellularisation, the peritoneum was incubated in 3.4% DOC dissolved in MQ water for 4 h at 4 °C on a tilting platform. After decellularisation, the peritoneum was washed in PBS^—^5 times for 1 h at 4 °C, followed by treatment with 0.167 mg/mL DNAse I dissolved in PBS^+^ for 2 h at 37 °C under continuous agitation. The peritoneum was then washed with PBS^—^3 times for 1 h at 4 °C on a tilting platform. Decellularised tissue samples for histopathological analysis, DNA quantification, and proteomics were collected as described above. The remaining decellularised scaffold was stored in PBS^—^ with 1% P/S at 4 °C. Generated PerMa scaffolds were g-sterilised with a dosage of 25 kGy (Gammatom srl, Guanzate, Italy) and stored in PBS^—^ with 1% P/S at 4 °C until further use.

### Histology and immunohistochemistry

Specimens from native organs, decellularised scaffolds, and scaffolds repopulated with cells were fixed with 4% PFA for 1 h at room temperature and embedded in paraffin. Formalin-fixed paraffin-embedded (FFPE) samples were cut into 3 μm thick sections.

#### Haematoxylin and eosin staining

Slides were deparaffinised in xylene for 20 min, rehydrated through 100%, 95%, and 70% ethanol baths (10 dips in each), and rinsed in water. Slides were incubated for 7 min in haematoxylin, then rinsed in water, dipped 8 times in 0.25% ammonia solution, rinsed for 5 min in water, incubated for 1 min in eosin, rinsed by 10 dips in water, and immediately dehydrated through 70%, 95%, 100% ethanol baths, and xylene before mounting.

#### Elastica/Van Gieson

Deparaffinised and rehydrated samples were stained with an Elastica Van Gieson staining kit (Roche Tissue Diagnostics, Tucson, AZ, USA): 4 min incubation in haematoxylin, followed by 12 min incubation in FeCl Oxidiser & Lugol Iodine, washing with tap water, 8 min incubation in Differentiator, washing with tap water, and finally 4 min incubation in Van Gieson CS, dehydration and mounting.

#### Alcian Blue

Deparaffinised and rehydrated samples were stained with an Alcian Blue Staining Kit (Roche Tissue Diagnostics, Tucson, AZ, USA): 8 min incubation in Alcian Blue, washing, 4 min incubation in Alcian Blue nuclear fast red (NFR), and finally washing, dehydration and mounting.

#### Immunohistochemistry (IHC)

Deparaffinization, antigen retrieval, and staining were done with a Dako PT-link, following the Dako Flex + protocol (Agilent Technologies, Santa Clara, CA, USA). Antigen retrieval was done with low or high pH, as detailed in Supplementary Table S1. Immunohistochemical staining was done with the antibodies listed in Supplementary Table S1. Sections were counterstained with haematoxylin.

### DNA quantification

Specimens of native organs and decellularised scaffolds were lyophilised overnight, weighed and chopped into 1 × 1 mm pieces, and digested with Proteinase K (Qiagen, Hilden, Germany) for 3 h at 56 °C. DNA was extracted using the DNeasy blood and tissue kit (cat# 69504, Qiagen, Hilden, Germany) according to manufacturer's instructions. DNA content was quantified with the Quant-iT™ PicoGreen™ dsDNA Assay Kit (ThermoFisher Scientific, Waltham, MA, USA) according to the manufacturer's instructions. Measurements were performed at an excitation of 480 nm and an emission of 520 nm using a SpectraMax Gemini EM plate reader (Molecular Devices, San Jose, CA, USA). DNA values were determined from a Lambda-DNA standard curve.

### 3D printed crowns

Culture plate inserts, referred to as crowns, and staining rings, were custom-designed and 3D printed with a Form 2 3D printer (Formlabs, Somerville, MA, USA), using High Temp resin (Formlabs, Somerville, USA). The dimensions are given in Supplementary Fig. S2, and stl-files are given as Supplementary Files. The post-processing included 15 min of soaking in isopropyl alcohol, followed by drying with compressed air to remove excess print material and curing in a UV chamber in two rounds of 20 min. The crowns and staining rings were autoclaved before use for cell cultures.

### Multiphoton microscopy

#### Sample preparation

Native organs were imaged on the day of sample collection. Decellularised scaffolds were imaged within one week after decellularisation. For imaging, scaffolds were mounted on 3D printed crowns and placed in PBS or cell culture medium (Fig. 3). The multiphoton objective was placed on top of the culture plate, and therefore, the crowns were placed in a reversed position in a 6-well plate for imaging.

#### Imaging

For each tissue type, samples from three to five donors were imaged. Three random fields for each sample were imaged with an Olympus FluoView FVMPE-RS > multiphoton laser scanning microscope equipped with an Ultrafast Ti:Sapphire Laser (Olympus, Tokyo, Japan). Images were captured in water immersion with a 25× objective at 920 nm excitation and 515–560 nm for intrinsic two-photon excitation fluorescence (TPEF) and 490–515 nm emission for second harmonic generation (SHG). Detector voltage was 400 V for TPEF and 450 V for SHG. The image field was 509 × 509 mm with a resolution of 1024 × 1024 pixels and a Z step size of 2 mm.

#### Image analysis

Image processing was performed in FIJI (National Institute of Health, Maryland, USA). Prior to image analysis, the images were cropped to 250 × 250 mm in four consecutive Z stacks of 10 mm depth starting at the surface of the tissue. Maximum projection images were generated from the Z stacks prior to further image analysis. Extracellular matrix patterns were analysed using the ImageJ plugin TWOMBLI (The Workflow Of Matrix BioLogy Informatics v.1, github. com/wershofe/TWOMBLI).^36^ The TWOMBLI parameters were: contrast saturation = 0.35, min line width = 1, max line width = 40, min curvature window = 5, max curvature window = 95, min branch length = 1, max display high-density matrix (HDM) = 255, and min gap diameter = 10. For each sample, the values for the three image positions were averaged.

### Diffusion assay

Diffusion assay was performed as previously described.^37^ In short, FITC-dextran with an average molecular weight of 4 kD or 40 kD (cat # 46944 and FD40, Sigma Aldrich, St. Louis, MO, USA) was dissolved in PBS^—^ at a concentration of 10 μM, and low-molecular-weight fragments were removed with Amicon Ultra-4 Centrifugal Filters with a cut-off of 3 kD or 30 kD (cat# UFC8003 and UFC8030, Merck Millipore, Burlington, VT, USA). Scaffolds were mounted on 3D printed crowns to generate a Transwell®-like system and incubated with PBS^—^ at 4 °C overnight. At the start of the experiment, 2200 μL fresh PBS^—^ was added to the outer compartment, and 1000 μL of 10 μM FITC-dextran solution was added to the inner chamber. The plate was placed in an incubator at 37 °C, 5% CO_2_ for the remainder of the experiment. At given time points (0, 5, 10, 20, 40, 60, 90, 120, 180, 240, 300, 360, 1440 min) 50 μL samples were taken from the outer compartment and the initial volume in the outer compartment was restored by addition of fresh PBS^—^. The fluorescence signal was measured at an excitation of 490 nm and an emission of 525 nm using a SpectraMax Gemini EM plate reader (Molecular Devices, San Jose, CA, USA). The amount of FITC-dextran that diffused across the scaffold over time was determined from a FITC-dextran standard curve. The apparent permeability coefficient (P_app_), giving the passive permeability of the substance per area over time, was calculated as described before.^38^ Briefly, P_app_ is calculated using the following formula:

$$P_{app}=\frac{\left(dQ/dt\right)}{\left(A\;\times \;C0\right)}$$

where dQ/dt is the rate of permeability (mmol/s), C_0_ is the initial concentration in the donor compartment, and A is the surface area of the filter (1.33 cm^2^).

### Rheology

Rheological measurements were conducted using an Anton Paar Physica MCR302 (Anton Paar, Ostfildern, Germany) in a plate to plate set up equipped with a 25 mm diameter measuring probe, a solvent trap and a Peltier element. Samples were trimmed to fit the probe diameter and soaked with PBS^—^. The gap width was determined by lowering the probe until a standard force of 1 N was reached. Measurements were performed at 37 °C. Initially, a frequency sweep from 0.1 to 100 rad/s was performed at 0.5% strain deformation, followed by an amplitude sweep with increasing strain deformation from 0.01 to 500% at an angular frequency of 10 rad/s. Mean values of storage and loss modulus as well as the damping factor were calculated from the linear region of amplitude sweep measurements. The flow point was determined as shear stress at the intersection point of storage and loss modulus (G’ = G’’).

### Proteomics

#### Cell lysis and protein digestion

Ten mg of lyophilised native tissues and dECM scaffolds were weighed and chopped into 1 × 1 mm pieces. The samples were lysed in lysis buffer (100 mM Tris–HCl, pH 7.6, 5% SDS in MilliQ water) and sonicated 3–6 cycles of 30 s with 30 s rest on ice at power 30% (Sonics VibraCell CP130 sonicator, Cole–Parmer Instruments, Vernon Hills, IL, USA). The samples were boiled for 7 min at 95 °C and centrifuged for 10 min at 14000×*g*. Protein lysates were transferred to new tubes. Protein concentration was measured using Pierce™ BCA Protein Assay Kit (cat# 23227, ThermoFisher Scientific, Waltham, MA, USA) according to the manufacturer's instructions.

#### On-beads precipitation and protein digestion

Ten μg of the samples were precipitated with 70% acetonitrile onto magnetic beads (MagReSynAmine, Resyn Biosciences, Gauteng, South Africa). The proteins were washed on the beads with 100% acetonitrile, and 70% ethanol and then resuspended in 100 μL 50 mM ammonium bicarbonate. The proteins were reduced by the addition of 0.5 M dithiothreitol (DTT) to a final concentration of 10 mM and incubation at 56 °C for 30 min. Then, to alkylate proteins, 2.7 μL of 550 mM iodoacetamide (IAA) was added to a final concentration of 15 mM, and samples were incubated at room temperature in the dark for 30 min. 0.5 μg trypsin (Promega, Madison, WI, USA) was added to each sample for overnight on-beads protein digestion at 37 °C. The resulting peptides were concentrated and desalted before mass spectrometry analysis using the STAGE-TIP method with C18 resin disk (Affinisep).

#### LC-MS/MS analysis

Samples were analysed by a nanoElute UHPLC coupled to a timsTOFfleX mass spectrometer (Bruker Daltonics, Bremen, Germany) via a CaptiveSpray ion source. Peptides were separated on a 25 cm reversed-phase C18 column (1.6 μm bead size, 120 Å pore size, 75 μm inner diameter, Ion Optics) with a flow rate of 0.3 μL/min and a solvent gradient from 0 to 35% B in 60 min. Solvent B was 100% acetonitrile in 0.1% formic acid and solvent A 0.1% formic acid in water. The mass spectrometer was operated in data-dependent Parallel Accumulation-Serial Fragmentation (PASEF) mode. Mass spectra for MS and MS/MS scans were recorded between *m*/*z* 100 and 1700. Ion mobility resolution was set to 0.85–1.30 V s/cm over a ramp time of 100 ms. Data-dependent acquisition was performed using 4 PASEF MS/MS scans per cycle with a near 100% duty cycle. A polygon filter was applied in the *m*/*z* and ion mobility space to exclude low *m*/*z*, singly charged ions from PASEF precursor selection. An active exclusion time of 0.4 min was applied to precursors that reached 20,000 intensity units. Collisional energy was ramped stepwise as a function of ion mobility.

#### LC-MS/MS data analysis

MS raw files were submitted to MaxQuant software version 2.4.7.0 for protein identification and label-free quantification.^39^ Parameters were set as follow: fixed modification: carbamidomethylation (C), protein N-acetylation and methionine oxidation as variable modifications. First search error window of 20 ppm and main search error of 4.5 ppm. Trypsin without proline restriction enzyme option was used, with two allowed miscleavages. Minimal unique peptides were set to 1, and false discovery rate (FDR) allowed was 0.01 (1%) for peptide and protein identification. For porcine samples the sus scrofa trembl and Swissprot (downloaded 2023) databases were used and for human samples the homo sapiens Swissprot database from uniprot (downloaded 2022) was used. The function match-between-runs were deactivated. Generation of reversed sequences were selected to assign FDR rates.

For statistical comparison analysis, Perseus version 2.0.11 was used. Intensities were log2 transformed and data was filtered based on 50% valid values in at least one group, missing values were imputed from normal distribution and student t-test was performed with permutation-based FDR cutoff of p-value <0.05. The limited availability of human samples from autopsies (hPerMa) and HGSOC patients (ocPerMa) constrained the statistical analyses. Statistical methods were selected to suit the available sample size, with a focus on quantitative comparisons between groups.

#### Matrisome matching

Protein ID from MaxQuant proteinGroup file was mapped to gene names using the uniprot protein ID mapping. Around 40% of the protein IDs could not be mapped. The gene names were matched to the matrisome database.^40^

#### Data availability

The mass spectrometry proteomics data have been deposited to the ProteomeXchange Consortium via the PRIDE partner repository with the dataset identifier PXD049045.^41^

### SIS and PerMa cell cultures

#### Cell culture

Scaffolds were mounted as a Transwell®-like system (Fig. 3C) and placed in 12-well plates in cell culture medium at 37 °C, 5% CO_2_ overnight. The following day, 1.5 mL medium was added to the outer chamber, and cells were seeded in the inner chamber in a volume of 1 mL. For SIS monocultures, ovarian cancer cells were seeded on the submucosal surface. For PerMa, cells were seeded on the layer facing the peritoneal cavity. COV318 and OV90 cells were seeded at 25,000 cells/scaffold, whereas Caov3 cells were seeded at 400,000 cells/scaffold for monoculture and 200,000 cells/scaffold for coculture. For cocultures, BJ fibroblasts were seeded in a number of 12,500 cells/scaffold (COV318), 6250 cells/scaffold (OV90), and 25,000 cells/scaffold (Caov3). The cells were cultured at 37 °C, 5% CO_2_. All EOC cell lines were GFP+ and the BJ fibroblasts were miRFP670+. For each cell line, the 21-day culture experiment was independently repeated twice. Within each experiment, three technical replicates were included per condition, and three areas were imaged in each technical replicate. PerMa scaffolds from different pigs were used in each experiment to account for biological variability.

#### Imaging

Scaffolds were imaged on days 3, 7, 10, 14 and 21 using a Dragonfly 505 confocal spinning disk system (Andor Technologies, Inc, Belfast, Northern Ireland). To enable imaging, scaffolds were flipped so that the cells were on the outer surface of the scaffold on day 3 and kept in that position for the remaining period of the experiment. During imaging, the crowns were moved to a 6-well plate with a droplet of cell culture medium, with the scaffold facing down towards the microscope objective (Fig. 6a). For each scaffold, three representative imaging fields were selected and imaged at 10× magnification, with a Z-stack depth of 150–300 μm and a 2 μm step size. The background matrix structure was imaged using a 561 nm laser line and a 575–625 nm filter. GFP + cells were imaged using a 488 nm laser line and a 500–550 nm filter. For cocultures on PerMa, BJ miRFP670+ fibroblasts were added to the culture and imaged using a 637 nm laser line and a 663–738 nm filter.

#### Image analysis

Image processing was performed in IMARIS software v. 9.9 (Bitplane, Zurich, Switzerland). Quantification of fluorescent signal was analysed using the surfaces function.

### Drug screen and viability assay

Carboplatin (cat # 507662, 10 mg/mL, Fresenius Kabi, Bad Homburg, Germany) was used within 48 h after the seal was broken. Drug dilution series were prepared using cell culture medium. OV90 cells were used for all drug sensitivity assays.

To determine carboplatin EC_50_ values for the OV90 cell line cultured in a 2D monolayer, WST-1 assay was performed (cat # 11644807001, Roche, Basel, Switzerland). 2000 cells/well were seeded in 96 well cell culture plates and allowed to attach for 24 h. The following day, the culture medium was removed, and carboplatin at different concentrations was added onto the plate. After 72 h, WST-1 reagent was added (1:10) to each well and incubated for 2 h at 37 °C, 5% CO_2_. The absorbance of formazan was measured at 440 nm with a Spectra Max Plus 384 microplate reader (Molecular Devices, San Jose, USA). The experiment was performed in triplicate and repeated three times.

Carboplatin sensitivity for OV90 in 2D culture was also assessed using apoptosis and necrosis as markers for cell viability. Five doses (10 μM, 31.6 μM, 45.6 μM (=EC50), 100 μM, and 316 μM) were chosen based on the WST-1 assay results. 90,000 cells/well were seeded in 6 well culture plates in 2 mL volume and allowed to attach for 24 h. The following day, the culture medium was removed, and carboplatin at different concentrations was added onto the plate at a volume of 3 mL per well. After 72 h, the medium from each well was collected in individual tubes. The remaining attached cells were then trypsinised and combined with the medium containing detached cells. The cells were stained with Annexin V/PI (cat #V13245, Invitrogen, Waltham, MA, USA) and analysed by flow cytometry for detection of apoptotic and necrotic cells. The experiment was performed with three technical replicates for each drug dose.

Based on the EC50 value for OV90 and the results from the apoptosis/necrosis assay, three drug doses (10 μM, 50 μM, and 100 μM) were chosen for further drug sensitivity testing in 3D culture. PerMa was mounted as a Transwell®-like system and placed in 12 well plates in cell culture medium at 37 °C, 5% CO_2_ overnight. The following day, 25,000 cells/scaffold were seeded. On day three after seeding, the scaffolds were flipped, leaving the cells on the outer surface. On day seven after seeding, the PerMa scaffolds were imaged using a Dragonfly 505 confocal spinning disk system (Andor Technologies). Following imaging, the scaffolds were moved to a new 12-well plate containing carboplatin at different doses and cultured at 37 °C, 5% CO_2_. On day ten after seeding, the PerMa scaffolds were stained with an apoptosis/necrosis assay kit (cat # ab176749, Abcam, Cambridge, United Kingdom). Staining was performed according to the manufacturer's recommendations using the 3D-printed staining ring. Briefly, the staining reagents and washing buffers were added in a volume of 300 μL to the staining ring, and the crowns with PerMa were placed on top of the staining ring. Staining was performed according to manufacturer's recommendations. Scaffolds were washed in washing buffer for 1 min, stained with 7-AAD, Apopxin Green, and CytoCalcein Violet for 30 min, followed by 3 × 1 min washing in washing buffer. The scaffolds were imaged using the Dragonfly confocal microscope. Images were taken at 10× magnification, with a Z-stack depth of 150–300 μm with a 2 μm step size. Three representative imaging fields were selected for each scaffold. 7-AAD was imaged using a 561 nm laser line and a 575–625 nm filter. Apopxin Green was imaged using a 488 nm laser line and a 500–550 nm filter. CytoCalcein Violet was imaged using a 405 nm laser line and a 425–475 nm filter. Image analysis was performed in the IMARIS image analysis software using the surfaces and spots functions.

### CAR T cytotoxicity testing

TAG72 CAR single chain variable fragment (scFv), huCC49, was derived from Kashmiri et al.^42^ Muc16 CAR scFv (K101) was generated as described in Casey et al.^43^ Both CARs are second-generation containing a CD8 hinge and CD8 transmembrane domain, a 4-1BB intracellular domain, and CD3ζ.^44^ Retroviral particles carrying the CAR vectors and transduction of T cells were done as described previously.^45^ Briefly, HEK cells were transfected with packaging vectors and a retroviral vector encoding the CAR constructs. After 2 days of harvesting, the supernatant, containing viral particles, was used in two rounds of transduction of PBMCs activated for 48 h with anti-CD3 and anti-CD28 antibodies (coated on a 24-well plate at 1 μg/mL respectively). CAR expression on the T cells was assessed with an anti-G4S linker (Cell Signalling Technology, US) 3 and 8 days after transduction.

Cell cultures were prepared as described in section 2.11. Briefly, 25,000 OV90 cells were seeded per scaffold and treated with CAR T cells targeting TAG-72 or Mock T cells. The scaffolds were flipped and imaged with a Dragonfly 505 confocal microscope after three and seven days to confirm cell growth. On day eight, the scaffolds containing OV90 cells were flipped back so the cells were again on the inner surface and placed in new wells containing 1.5 mL medium. CAR T or mock T cells were labelled with CellTrace™ Violet Cell Proliferation Kit (CTV; cat #C34557, Invitrogen, Waltham, USA) according to the manufacturer's protocol and seeded in a volume of 0.5 mL to the inner chamber. The scaffolds were incubated at 37 °C, 5% CO_2_. After 24 h, the scaffolds were flipped, so the cells were again on the outer surface and imaged with a Dragonfly 505 confocal microscope, as described above. CAR T/mock T cells were imaged using a 405 nm laser line and a 425–475 nm filter and GFP positive EOC cell lines using a 488 nm laser line and a 500–550 nm filter. Image analysis was performed in the IMARIS image analysis software using the surfaces function.

### Statistics

Statistical analyses were performed in PRISM Graphpad (version 10.1.0). Data is presented as mean ± standard deviation. For analyses comparing two datasets, unpaired t test was used. For comparison of more than two datasets, analysis of variance (ANOVA) with Tukey's multiple comparison test was used. Significance was set to p < 0.05. When data allowed, we assessed normality using the Shapiro–Wilk test and confirmed normality before applying parametric methods. For datasets with small sample sizes (below n = 3), formal normality testing lacks sufficient power. Details about statistical analyses, including p values, are presented in the figure legends. No formal sample size calculation was performed. For the human tissue, sample size was constrained by ethical and logistical factors, including strict inclusion/exclusion criteria for autopsy material and limited availability of patient-derived samples with informed consent. Within these constraints, all available samples were included, and appropriate statistical methods were applied to account for group sizes. For the animal tissue, the number of animals used was determined by the amount of tissue required to generate sufficient decellularized scaffolds for all *in vitro* experiments and to adjust for biological sample variation.

### Role of funders

Financial support was provided by the University of Bergen, Helse Vest RHF (F-12183-D10616, 779, 911182, 912035, and 912146), Helse Bergen HF (240222), the Norwegian Cancer Society (6833652 and 182735), the Research Council of Norway grants (250317, 326300, 223250, 262652, and 295910), the Novo Nordisk Foundation (NNF21OC0070381), the Kolbjørn Brambani Legat for Kreftforskning, the National Institute of Health (R01CA199646) and the Swedish Cancer Society (21 1888 Pj). HF (240222), the Norwegian Cancer Society (6833652 and 182735), the Research Council of Norway (FRIPRO program (project numbers 250317, 326300, 223250, and 262652) and INFRASTRUKTUR program (295910), the Centre for Cancer Cell Reprogramming (CanCell), the Novo Nordisk Foundation (NNF21OC0070381), the Kolbjørn Brambani Legat for Kreftforskning, the National Institute of Health (R01CA199646) and the Swedish Cancer Society (21 1888 Pj). Funding sources were not involved in the writing of the manuscript. We were not paid to write this article by a pharmaceutical company or other agency.

## Results

### Chemical and enzymatic decellularisation of peritoneum results in a cell-free ECM scaffold

**Fig. 1:**
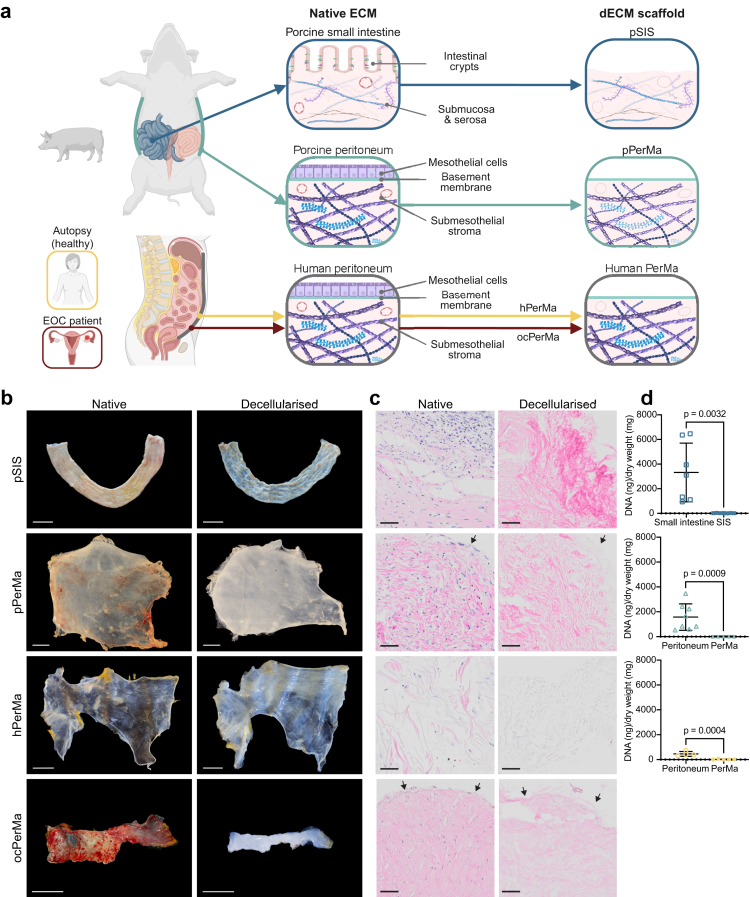
**Decellularisation of small intestine and peritoneum generates dECM scaffolds. a** Schematic illustration of the generation of dECM scaffolds from porcine small intestine and peritoneum, and human peritoneum. **b** Photographs of porcine small intestine and porcine and human peritoneum before (left) and after (right) decellularisation. Scale bars 30 mm. **c** Corresponding haematoxylin and eosin (H&E) staining of native and decellularized tissues. Scale bars 50 μm. Arrows show tissue surface where the mesothelial cell layer is present in native and absent in decellularized tissue. **d** DNA content of native and decellularized porcine small intestine (n = 7 native and n = 7 decellularized), porcine peritoneum (n = 8 native and n = 8 decellularized), and human peritoneum (n = 4 native and n = 6 decellularized). Data represent mean ± SD, unpaired two-tailed t-test.

3D *in vitro* organotypic models can be generated by culturing normal and malignant cells on decellularised tissue scaffolds, such as the porcine small intestinal submucosa (SIS) scaffold.^31^^,^^32^ To establish a model that mimics peritoneal HGSOC metastases, we developed a decellularised tissue scaffold from porcine peritoneum, referred to as pPerMa. We compared this to the previously established porcine SIS (pSIS) and decellularised human peritoneum obtained from either autopsy of patients with no intra-abdominal disease (hPerMa) or surgery of patients with EOC (ocPerMa; Fig. 1a).

Porcine and human parietal peritoneum were collected from the anterior abdominal walls and decellularised using a chemical detergent followed by enzymatic DNA degradation (Supplementary Fig. S1). We chose the detergent sodium deoxycholate (SDC) since it has been shown to effectively decellularise tissues of various thicknesses without compromising the structure of the ECM.^46^, ^47^, ^48^ The SIS scaffold was prepared as described previously.^35^

To evaluate the decellularisation efficacy, we assessed cell removal by histology and residual DNA content. Samples were taken from native intestine and peritoneum as well as from small intestinal submucosa (SIS) and peritoneal matrix (PerMa; Fig. 1b). The decellularisation resulted in a cell-free scaffold as demonstrated by haematoxylin and eosin (H&E) staining (Fig. 1c). DNA quantification confirmed depletion of cell nuclei and genetic material to an average 20.03 ± 10.97 ng/mg in pSIS, 2.91 ± 4.62 ng/mg in pPerMa, and 12.48 ± 26.95 ng/mg in hPerMa (Fig. 1d). Altogether, the decellularisation protocol results in an efficient removal of cells and a significant decrease in DNA content.

### A custom-designed culture plate insert enables characterisation and cell culture

**Fig. 2:**
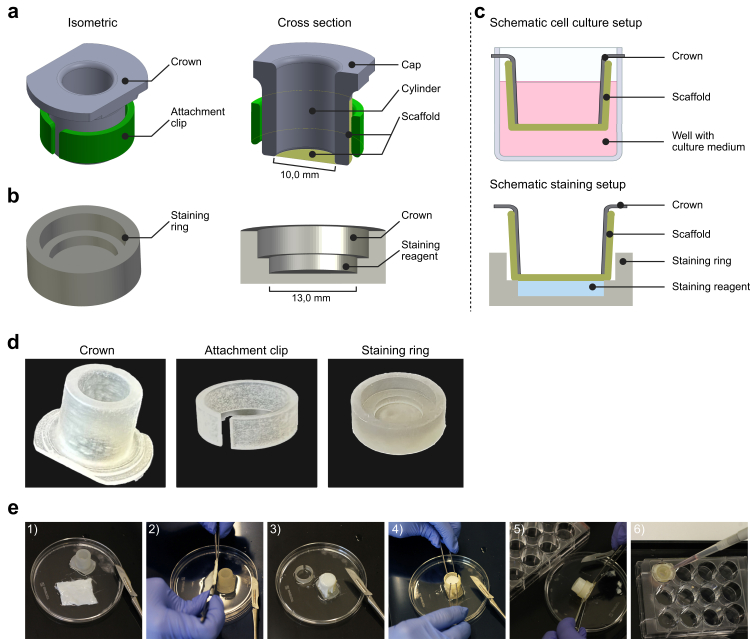
**Custom-designed 3D-printed culture plate inserts and staining rings enable cell culture, staining, and confocal imaging of 3D cultures using dECM scaffolds. a** A 3D-printed culture plate insert, referred to as a crown, suspends and secures the dECM scaffold above the surface of a culture plate well, creating a Transwell®-like setup. **b** A staining ring enables staining of the dECM scaffold in a volume of 300 μL. **c** Schematic showing how the scaffold is fixed to the crown and suspended in a 12-well plate during cell culture (top), and how the crown with scaffold is placed on the staining ring during staining (bottom). **d** Photographs of the crown, the attachment clip, and the staining ring. **e** Visualisation of the sequential steps in scaffold preparation. 1) The tissue is cut into appropriately sized tissue sections to fit the crown. 2) The tissue section is placed over the crown, and 3) stretched across. 4) The attachment ring is placed on top of the scaffold to secure the scaffold to the crown. 5) Excess tissue is removed with a scalpel, and the crown is placed in a 12-well plate containing medium. 6) Medium is added to the inside of the crown.

To enable the characterisation and the establishment of cell cultures with the dECM scaffolds, we engineered a 3D printed culture plate insert, referred to as a crown with attachment clip (Fig. 2a, d and Supplementary Fig. S2a and b). The crown serves three key functions: 1) elevating and securing the dECM scaffold above the surface of a multiwell culture plate, 2) establishing a Transwell®-like system where two chambers are separated solely by the scaffold, and 3) enabling confocal imaging, ensuring direct contact between the scaffold and the imaging surface. To enable the staining of scaffolds in a lower volume, we developed a 3D-printed staining ring (Fig. 2b, d, Supplementary Fig. S2c). The crowns and staining ring were developed to fit 12-well plates.

Our design centred on a cylindrical structure, optimising gas and fluid access to the dECM scaffold using conventional pipetting techniques. The inner chamber has a diameter of 10 mm, complemented by a wall thickness of 2.5 mm (Fig. 2a, Supplementary Fig. S2a). This thickness was strategically chosen based on the material's properties, balancing the ease of printing and processing with durability for repeated use. To prevent the dECM scaffold from contacting the bottom of the well, we incorporated a cap with a larger diameter (24 mm), exceeding the well diameter of the 12-well plate (Fig. 2a, Supplementary Fig. S2a). This design prevents interference with adjacent wells. To ensure the crown's centred positioning within the well, we added a notch in the cap at a diameter of 20.5 mm (0.5 mm smaller than the well's diameter; Supplementary Fig. S2a). The main cylinder's depth was set at 13.00 mm to avoid contact with the well bottom. Where feasible, we added fillets of 1 mm or 0.5 mm to mitigate any potential damage to the dECM scaffold during application.

A critical aspect of the design was a small “attachment clip,” functioning similarly to an open C-clip (Fig. 2a, Supplementary Fig. S2b). This clip leverages the flexibility of the 3D printed material to exert sufficient clamping force on the dECM scaffold, ensuring secure retention while allowing for easy removal without damage to the dECM scaffold. The clip's wall thickness is 1.5 mm, fine-tuned for optimal clamping force. To further protect the dECM scaffolds, the retaining clip includes fillets of 0.5 and 1.0 mm to prevent puncturing during application.

The staining ring has a chamber with a volume of 300 μL that is filled with staining solution. The crown is placed on top of this chamber, supported on the sides.

FormLabs High Temp Resin (FLHTAM01) can withstand autoclave temperatures and has previously been demonstrated to be biocompatible after post-print treatment.^49^^,^^50^ We did not observe adverse effects of the material on cell viability.

### The dECM scaffolds retain the structure of the native tissues and show similarities in structure

**Fig. 3:**
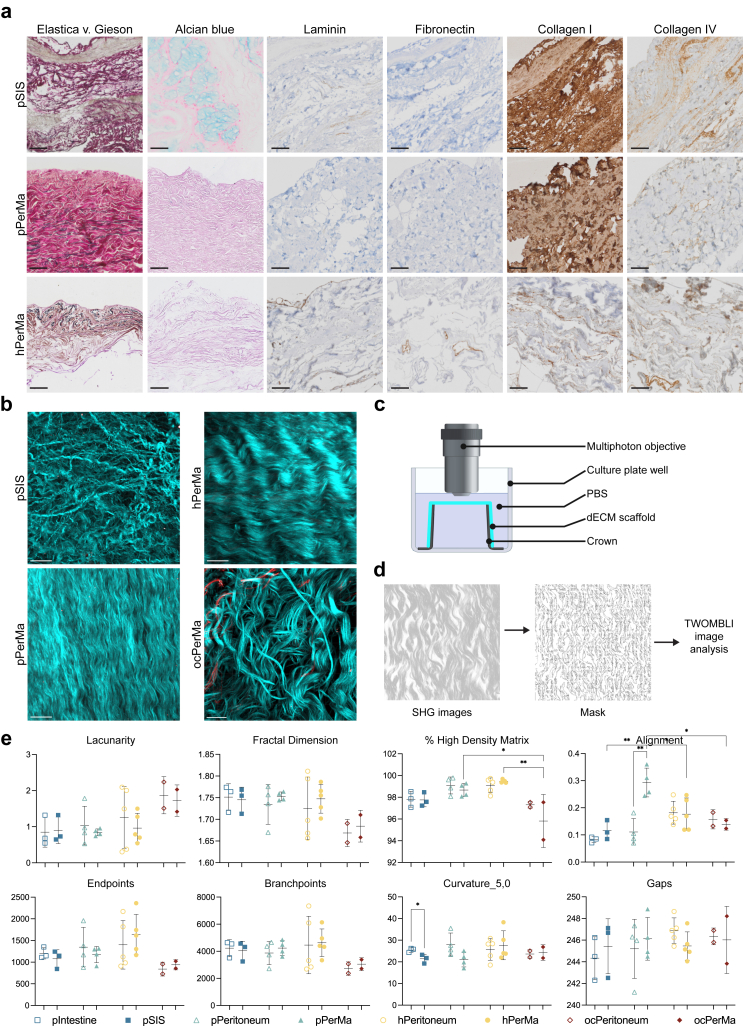
**The structure of major ECM proteins differs between the dECM scaffolds. a** Representative images of Elastica v. Gieson and Alcian blue staining and immunohistochemical staining of laminin, fibronectin, collagen I, and collagen IV in pSIS, pPerMa, and hPerMa. Scale bars 50 μm. **b** Representative second harmonic generation (SHG; cyan)/two-photon excitation fluorescence (TPEF; red) microscopy images of pSIS, pPerMa, hPerMa, and ocPerMa. 3D renderings (left) and single optical sections (right). Scale bars = 50 μm. **c** Schematic illustration of the setup for multiphoton imaging. The dECM scaffolds are mounted on crowns and placed bottom-up in a 6-well plate filled with PBS. The multiphoton objective is immersed in PBS. **d** Example images showing the masking of SHG images for The Workflow Of Matrix BioLogy Informatics (TWOMBLI) image analysis. **e** TWOMBLI analysis results. Data represent mean ± SD. ∗p < 0.05, ∗∗p < 0.01. One-way ANOVA with Tukey's multiple comparison test was used for comparison of the decellularised matrices. Unpaired two-tailed t-tests were used for comparison of each pair of native/decellularised tissue. N = 3 for pIntestine/pSIS, n = 4 for pPeritoneum/pPerMa, n = 5 for hPeritoneum/hPerMa, n = 2 for ocPeritoneum/ocPerMa. Three representative images were captured and analysed for each sample. Images were cropped to 250 μm (x) ∗ 250 μm (y) ∗ 10 μm (z) stacks. Four z stacks were generated per image area to include the top 40 μm from the surface.

To assess the integrity of the ECM after tissue decellularisation, we compared histological staining of major ECM components and the structure of the ECM with multiphoton microscopy (Fig. 3 and Supplementary Fig. S3). Histological staining showed similar patterns of laminin, fibronectin, collagen I, and collagen IV positivity in the native (Supplementary Fig. S3a) and decellularised (Fig. 3a) tissues from the same source. Second harmonic generation (SHG) and two-photon excitation fluorescence (TPEF) by multiphoton microscopy allow label-free visualisation of non-centrosymmetric structures (typically collagen fibres) and tissue autofluorescence, respectively (Fig. 3b, Supplementary Fig. S3b).^51^ For multiphoton imaging, the scaffolds were mounted on crowns, placed in 6 well plates with the tissue facing up, and submerged in PBS (Fig. 3c). To assess the effect of decellularisation on the matrix patterns, we used the ImageJ plugin TWOMBLI to quantitatively analyse the multiphoton images of our samples (Fig. 3d).^36^ Fibre lacunarity, fractal dimension, % high-density matrix (HDM), branchpoints, and endpoints all remained unaltered between native and decellularised samples of the same tissue type (Fig. 3e). Only matrix alignment increased in the decellularised pPerMa (0.2931 ± 0.0531) compared to the native porcine peritoneum (0.1109 ± 0.0492), p = 0.0003 and curvature decreased in the decellularised pSIS (21.41 ± 2.011) compared to the native porcine intestine (25.44 ± 0.9989), p = 0.0358 (Fig. 3e). In summary, the decellularisation preserved the structure of major ECM components.

We also used histological staining and multiphoton microscopy to assess the differences between dECM scaffolds derived from different tissues. Alcian Blue staining is seen in the pSIS model whereas the pPerMa and the hPerMa models are negative (Fig. 3a). Laminin is expressed in vessels in the pSIS model, is absent in the pPerMa models, and stains vessels and the surface basal membrane in the hPerMa model. Fibronectin staining is negative in the pSIS and the pPerMa models, whereas vessels are stained in the hPerMa model. Collagen I staining is strongly and diffusely positive in the pSIS and the pPerMa models, and is more focal, partly in vessels, in the hPerMa model. Collagen IV staining is seen in both stroma and vessels in the pSIS model, whereas it is predominantly localised to vessels in the pPerMa and the hPerMa models. Elastica/Van Gieson stain demonstrated elastic fibres interspersed between collagen fibres in the submesothelial stroma of human and porcine PerMa. The elastic fibres of pSIS were located deeper in the tissue (Fig. 3a). Of note, the content of high-density matrix, as assessed by TWOMBLI in the multiphoton images, was significantly lower in ocPerMa (95.82% ± 2.440) compared to hPerMa (99.47% ± 0.140, p = 0.0005) and pPerMa (98.67% ± 0.599, p = 0.0101) (Fig. 3e). Instead, the matrix had significantly more fibre alignment in pPerMa (0.2931 ± 0.0531) compared to pSIS (0.1163 ± 0.0353, p = 0.0047), hPerMa (0.1765 ± 0.0595, p = 0.0273) and ocPerMa (0.1388 ± 0.0211, p = 0.0238) (Fig. 3e). No significant differences were observed between the dECM scaffolds in lacunarity, fractal dimension, curvature, branchpoints, or endpoints (Fig. 3e). Overall, the structure of major ECM components was most comparable in the dECM scaffolds from different normal tissue sources. Of note, the ocPerMa displayed a reduced high-density matrix compared to the others, however it should be noted the small number of just two tissues.

### PerMa is a viscoelastic, permeable scaffold

Next to structure, the physical properties of the ECM, such as elasticity or porosity are known to influence cellular behaviours, including spreading, growth, proliferation, migration, and differentiation.^52^ Therefore, we investigated whether the scaffolds differ in their physical characteristics. Oscillatory rheological measurements were conducted to assess the mechanical properties of the pPerMa in comparison to the pSIS and the hPerMa.

**Fig. 4:**
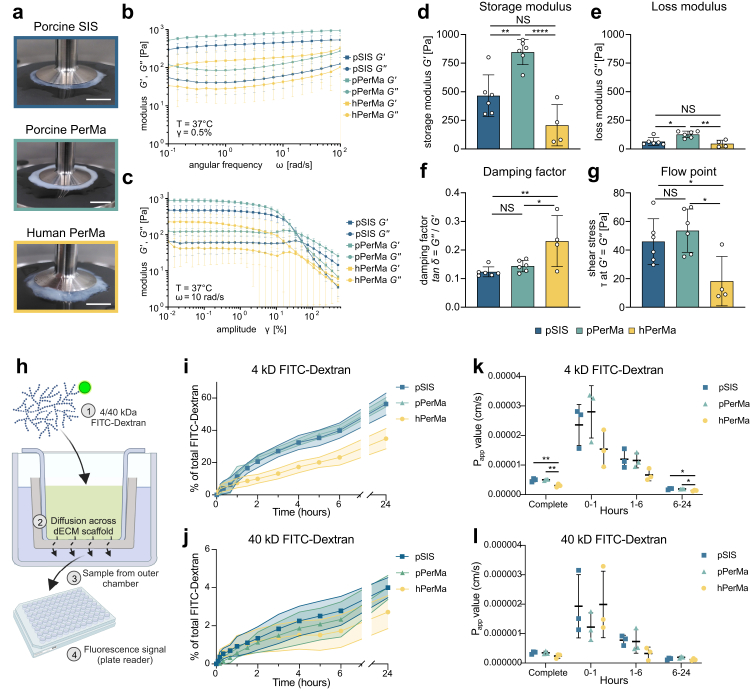
**Rheological analyses reveal differences in the stiffness and permeability of the dECM scaffolds. a** Photographs of porcine SIS (blue), porcine PerMa (green), and human PerMa (yellow) mounted in the plate-to-plate rheometer. **b**, **c** Data plot of oscillatory measurements showing the storage (G′, square) and loss (G″, circle) modulus of the pSIS, the pPerMa, and the hPerMa. Moduli are shown as a function of frequency (b, γ = 0.5%) and strain deformation (c, ω = 10 rad/s). **d–g** Rheological parameters of the dECM scaffolds calculated from the linear region of the amplitude sweep. Single data points represent measurements of individual biological samples. Data represent mean ± SD. ∗p < 0.05, ∗∗p < 0.01, ∗∗∗∗p < 0.0001, one-way ANOVA with Tukey's multiple comparison test. **h** Schematic illustration of the diffusion assay. The dECM scaffolds were mounted in a Transwell®-like setup. 4 or 40 kD FITC-Dextran was added to the inner chamber, and samples were taken from the outer chamber at different time points (0, 5, 10, 20, 40, 60, 90, 120, 180, 240, 300, 3600, and 1440 min). The fluorescence signal was analysed with a plate reader. **i**, **j** Diffusion of 4 kD (i) and 40 kD (j) FITC-Dextran across the scaffolds over time. Data represent mean ± SD displayed as error shadows. The experiment was performed three times with three replicates for each scaffold. **k**, **l** The apparent permeability coefficient (P_app_) for 4 kD (j) and 40 kD (k) FITC-Dextran calculated for different time frames. ∗p < 0.05, ∗∗p < 0.01, one-way ANOVA with Tukey's multiple comparison test.

Frequency sweep measurements revealed a similar response of all three scaffolds to increasing angular frequencies (Fig. 4b). Storage modulus (G’), which is a measure of the elastic properties of a material, and loss modulus (G’’), describing the viscous material portion, exhibited parallel runs, confirming a viscoelastic solid behaviour of all scaffolds. Shear moduli G’ and G’’ differed among the scaffolds (hPerMa < pSIS < pPerMa), indicating different viscoelastic properties. Strain sweep measurements confirmed this presumption (Fig. 4c). The pPerMa exhibited a significantly higher storage and loss modulus (846 ± 100 Pa and 122 ± 26 Pa) compared to the pSIS (464 ± 167 Pa and 60 ± 31 Pa) and the hPerMa (207 ± 156 Pa and 41 ± 27 Pa), demonstrating that the pPerMa is more rigid than hPerMa and the pSIS (Fig. 4d and e). Next, the damping factor (tan *δ* = G’’/G’) was calculated to assess the elastic and viscous proportions of the scaffolds. The pPerMa (0.14 ± 0.02) and the pSIS (0.12 ± 0.02) exhibited a significantly lower damping factor compared to the hPerMa (0.23 ± 0.08) (Fig. 4f), suggesting an increased portion of elastic components in the pPerMa. This was corroborated by analysis of the flow point, defining the transition from solid to viscous behaviour (G’ = G’’) of a viscoelastic material. For both, pPerMa (53 ± 14 Pa) and pSIS (46 ± 15 Pa), a significantly higher shear stress (τ) was required to reach the flow point compared to the hPerMa (18 ± 15 Pa) (Fig. 4g), indicating that both, pPerMa and pSIS contain more elastic components than the hPerMa. Summarised, the pPerMa is more rigid than the pSIS and the hPerMa while the viscoelastic behaviour is more similar to the porcine SIS.

To evaluate scaffold permeability, we analysed the diffusion of molecules with low (4 kDa) and high (40 kDa) molecular weights across the scaffolds. Decellularised scaffolds were mounted in a Transwell®-like setup with an inner and an outer chamber separated only by the scaffolds (Fig. 4h). 4 kDa or 40 kDa FITC-Dextran were added to the inner chamber, and samples were taken from the outer chamber at different time points. Diffusion of FITC-Dextran across the scaffold was calculated as a percentage of total FITC-Dextran. The volume of the outer chamber was 68.75% of the total volume. All three matrices were permeable to low molecular weight FITC-Dextran (Fig. 4i). At 24 h, significantly less 4 kDa FITC-Dextran had diffused across hPerMa, compared to pSIS and pPerMa (34.83% vs. 56.33% and 57.45%, respectively). The diffusion rate, given by the apparent permeability coefficient (P_app_), was significantly lower for the hPerMa compared to pSIS and pPerMa for the complete period (p = 0.0078 and p = 0.0061, respectively; Fig. 4k). The matrices were only partially permeable to high molecular weight FITC-Dextran (Fig. 4j). At 24 h, 4.00% for pSIS, 4.01% for pPerMa, and 2.72% for hPerMa of 40 kDa FITC-Dextran diffused, with no statistically significant difference in amount diffused FITC-Dextran or P_app_ value. For both low and high molecular weight FITC-Dextran, the diffusion rate was highest within the initial 60 min (Fig. 4k, l). Interestingly, diffusion of 4 kD across the human PerMa was slower compared to the pPerMa and pSIS, indicating a lower permeability of the hPerMa.

### The dECM scaffolds have unique matrisome compositions

To characterise the biomolecular composition of the native tissues and decellularised scaffolds, we performed label-free quantitative proteomics by liquid chromatography-tandem mass spectrometry (LC-MS/MS). The number of identified proteins varied between the groups ranging from approximately 2000 to 7000 proteins and was consistently lower in the dECM scaffolds compared to the corresponding native tissues (Supplementary Fig. S4a). This is expected after the removal of cellular content.^53^ The number of identified proteins was significantly higher in pSIS compared to pPerMa, hPerMa, and ocPerMa (Supplementary Fig. S4b).

**Fig. 5:**
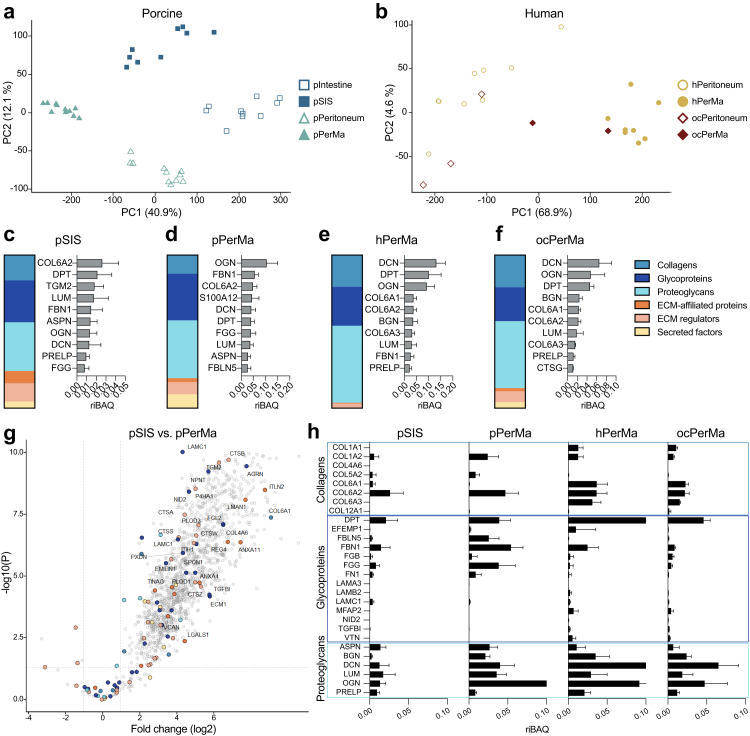
**Proteomic analysis by liquid-chromatography mass spectrometry (LC-MS/MS). a, b** Principal component analysis (PCA) plots of the total proteome for porcine (a) and human (b) samples. **c–f** Composition of total matrisome proteins and the ten most abundant matrisome proteins in pSIS (n = 10), pPerMa (n = 11), hPerMa (n = 8), and ocPerMa (n = 2). The ratios of the top ten proteins are given as iBAQ intensity relative to the total iBAQ intensity (riBAQ) for each sample. Data represent mean ± SD. **g** Volcano plot of protein intensities in pSIS (n = 10) vs. pPerMa (n = 11). Matrisome proteins with at least a 3-fold change (log2) are labelled. **h** Profile bar charts for pSIS (n = 10), pPerMa (n = 11), hPerMa (n = 8), and ocPerMa (n = 2) of selected core matrisome proteins (n = 28). The proteins are sorted into collagens (top box), glycoproteins (middle box), and proteoglycans (bottom box). The ratios are given as relative iBAQ intensities. Data represent mean ± SD.

Principal component analysis (PCA) showed that the biological replicates were similar (Fig. 5a and b). Both native and decellularised replicates clustered into separate groups, showing the reproducibility of the decellularisation method. While the native human peritoneum showed variation between replicates, the biological replicates of decellularised hPerMa were more similar.

To evaluate whether key ECM proteins were retained after decellularisation, we compared the abundance of core matrisome proteins and matrisome-associated factors, as defined by Naba et al., in native and decellularised samples.^40^ The relative abundance of matrisome and matrisome-associated proteins vs. non-matrisome proteins was higher in all dECM scaffolds compared to their native counterparts (Supplementary Fig. S4c). The proportion of core matrisome and matrisome-associated proteins increased from 12% in pIntestine to 18% in pSIS, from 27% in pPeritoneum to 57% in pPerMa, from 28% in hPeritoneum to 66% in hPerMa, and from 13% in ocPeritoneum to 45% in ocPerMa. Concordance was observed between native and decellularised tissues when comparing the 10 most abundant matrisome proteins (Fig. 5c–f, Supplementary Fig. S4d). Volcano plots comparing native and decellularised tissues revealed enrichment of particularly core matrisome proteins in the dECM scaffolds. However, there was also a depletion of several matrisome proteins, especially matrisome-associated proteins (Supplementary Fig. S4e–g). In summary, the decellularisation resulted in the enrichment of matrisome proteins and retained the most abundant ECM proteins.

We then compared the different dECM scaffolds. The composition of matrisome and matrisome-associated proteins was similar across tissues, with some organ-specific differences (Fig. 5c–f). All dECM scaffolds were rich in glycoproteins and proteoglycans. The highest proportion was seen in hPerMa, where glycoproteins and proteoglycans together constituted 76% of the total matrisome proteins (Fig. 5e). This proportion was 59% for pSIS, 68% for pPerMa, and 66% for ocPerMa. The samples displayed great diversity in the composition of different categories of matrisome proteins. Collagen Type VI (COL6A1-3), glycoproteins such as dermatopontin (DPT), and proteoglycans such as lumican (LUM) and decorin (DCN) were found among the top 10 most abundant matrisome proteins. Several of these were identified in the top 10 lists across all groups. Despite the similarities in matrisome composition across all dECM scaffolds, the volcano plot comparing pSIS and pPerMa revealed differences in the abundance of non-matrisome and matrisome proteins (Fig. 5g). A panel of 28 core matrisome proteins revealed further differences in the relative abundance between the dECM scaffolds (Fig. 5h).

In summary, porcine PerMa represents a biologically relevant tissue scaffold that shares several structural, physical, and biomolecular features with human PerMa, while also exhibiting some notable differences. Multiphoton imaging revealed key similarities in collagen organisation between hPerMa and pPerMa. Proteomic analysis showed that both scaffolds were rich in glycoproteins and proteoglycans, with comparable matrisome profiles overall, although hPerMa had the highest proportion of glycoproteins and proteoglycans. However, differences in mechanical properties and permeability were observed: pPerMa was more rigid than both hPerMa and pSIS, while hPerMa was the least permeable. Compared to other used scaffolds such as porcine SIS, pPerMa more closely resembles hPerMa in terms of ECM composition and organisation, supporting its potential as a suitable model for human peritoneal tissue.

### The pPerMa and the pSIS enable 3D in vitro cell cultures

Porcine tissues have several advantages over human tissues, including availability and less batch-to-batch variation. Therefore, porcine scaffolds were used to establish three-dimensional (3D) *in vitro* cell cultures. We first investigated the cytocompatibility of pSIS and pPerMa, and tested next, whether the ECM-specific differences in mechanical properties and protein composition of pSIS and pPerMa affected cellular behaviour *in vitro*.

**Fig. 6:**
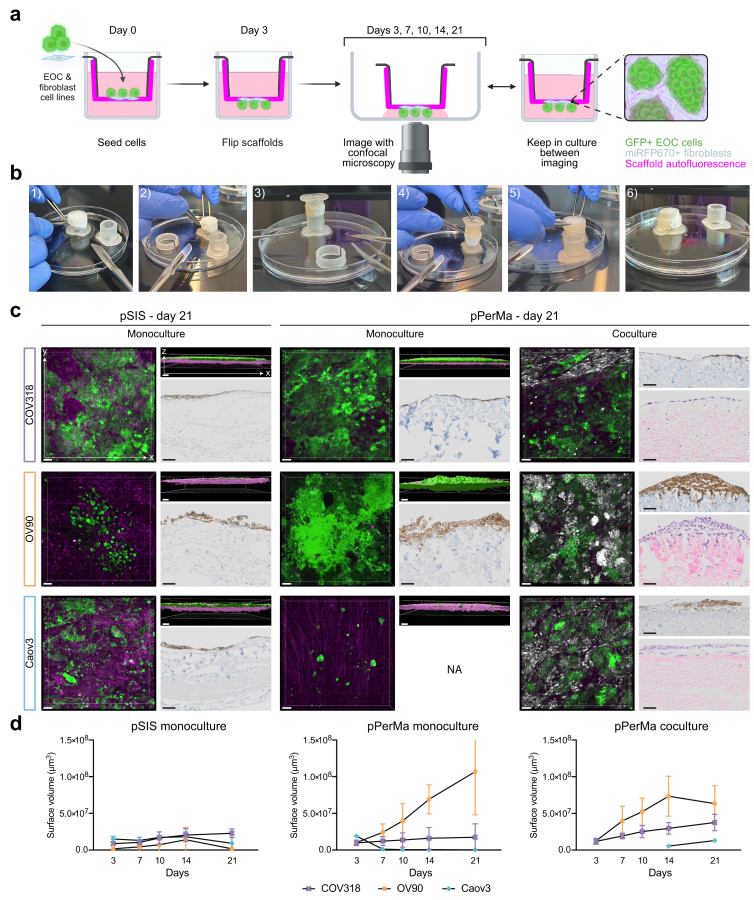
**3D cell cultures are established with the porcine dECM scaffolds. a** Schematic illustration of the setup for cell culture. Scaffolds were mounted in a Transwell®-like setup and cells were seeded in the inner chamber. After three days, the scaffolds were flipped, allowing the cells to come in direct contact with the imaging surface, enabling confocal imaging. Scaffolds were kept in this reversed position in the culture plate between imaging. **b** Photographs showing how the scaffolds are flipped. 1) A scaffold with cells cultured on the inside is placed in a Petri dish. 2) The attachment clip is removed. 3) The crown with the scaffold is placed on top of a new crown. 4) The scaffold is transferred to the new crown, leaving the cells on the outside of the crown. 5,6) A new attachment clip is placed on the crown and the crown is transferred back to the 12-well plate. **c** Confocal images and representative images of histological staining of cell cultures on pSIS and pPerMa. Each row represents a cell line (COV318, OV90, and Caov3). COV318 and OV90 cells were seeded in a number of 25,000 cells/scaffold, while Caov3 cells were seeded at 400,000 cells/scaffold for monoculture and 200,000 cells/scaffold for coculture. For cocultures, BJ fibroblasts were seeded in a number of 12,500 cells/scaffold (COV318), 6250 cells/scaffold (OV90), and 25,000 cells/scaffold (Caov3). Confocal images are 3D renderings of z-stacks, visualised in the x/y plane (left, quadratic images) and the x/z plane (right, rectangular images). Magenta represents tissue autofluorescence (ex = 561 nm, em = 575–625 nm), green represents GFP + EOC cell lines (ex = 488 nm, em = 500–550 nm), and white represents miRFP670+ BJ fibroblasts (ex = 637 nm, em = 663–738 nm). Scale bars = 100 μm. Histological stainings were performed on a cross-section of the scaffolds, corresponding to the x/z plane in the confocal images. The monocultures are stained with EpCAM. Cocultures are stained with EpCAM (top) and haematoxylin & eosin (H&E; bottom). Scale bars = 50 μm. NA = not applicable, too few cells to stain. The experiment was performed two times with three replicates for each group. **d** Graphs showing the cell growth of COV318, OV90, and Caov3 cells on pSIS (monoculture) and pPerMa (monoculture and coculture with fibroblasts). The cell volume was estimated using the surfaces function in the IMARIS image analysis software. Data represent mean ± SD.

The matrices were mounted on the crowns, and cells were seeded on the surface of the inner chamber (Fig. 6a and b). Three EOC cell lines—OV90, Caov-3, and COV318—were chosen based on their genetic resemblance to HGSOC.^54^ After three days, the scaffolds were flipped, leaving the cells on the outer surface where they were accessible for confocal imaging (Fig. 6a and b). The scaffolds were imaged on days 3, 7, 10, 14, and 21 after seeding (Fig. 6a). The surfaces function in the IMARIS image analysis software was used to estimate cell volume based on the fluorescent signal.

Both pSIS and pPerMa were cytocompatible and allowed cell growth. The three cell lines displayed distinct growth patterns. COV318 cells formed a monolayer on the surface of both pSIS and pPerMa, with similar growth kinetics (Fig. 6c and d, Supplementary Fig. S5a). There was no significant difference in cell volume between pSIS and pPerMa at any of the time points. OV90 cells formed superficial three-dimensional tumour-like structures, with an increased growth on pPerMa compared to pSIS (Fig. 6c and d, Supplementary Fig. S5b). The difference was statistically significant at all time points (p values 0.0001 day 3, 0.0032 day 7, 0.0101 day 10, 0.0004 day 14, and 0.0014 day 21 for unpaired, two-tailed t-test). Caov3 cells seeded in the same number as COV318 and OV90 did not grow, neither on the pSIS nor the pPerMa. When seeded at 400,000 cells/scaffold instead of 25,000, some cell growth was observed on pSIS but not on pPerMa (Fig. 6c and d, Supplementary Fig. S5c).

Several studies have found that cancer-associated fibroblasts (CAFs) promote peritoneal metastasis of EOC by contributing to adhesion and invasion.^55^, ^56^, ^57^ Therefore, we incorporated the human fibroblast cell line BJ in the 3D cell cultures on pPerMa. The BJ cell line was transduced with miRFP670, a far-red fluorophore, permitting simultaneous confocal imaging of GFP-positive tumour cells and fibroblasts. Interestingly, we found that Caov3 cells, which were unable to grow on pPerMa in monoculture, did grow in coculture with fibroblasts (Fig. 6c and d, Supplementary Fig. S6c). Moreover, the tumour cells formed clusters surrounded by fibroblasts, a pattern that is also seen *in vivo.*^58^ COV318 cells in coculture with fibroblasts grew as a monolayer separated from the fibroblasts, while OV90 cells grew interspersed with fibroblasts (Fig. 6c, Supplementary Fig. S6a and b).

### The 3D cell cultures can be used for therapy sensitivity testing

To assess sensitivity to therapy in the 3D *in vitro* model, we developed a method to assess cell death using confocal microscopy. For the establishment of the technique, we treated the cells with carboplatin, a chemotherapeutical used in the treatment of EOC. To select carboplatin doses to be tested in the 3D system, we determined the EC_50_ value of carboplatin for the OV90 cell line in 2D culture by WST-1 assay (Supplementary Fig. S7a). The cells were treated for 72 h with carboplatin at doses ranging from 1 μM to 3160 μM and an EC_50_ value of 45.60 μM for OV90 cells was determined. To validate the EC_50_ value, we performed a set of experiments to evaluate cell death by Annexin V/PI staining and flow cytometry in 2D cell culture (Supplementary Fig. S7b and c). The cells were treated for 72 h with carboplatin doses ranging from 10 μM to 316 μM, including the EC_50_ dose of 45.6 μM. The cells displayed increasing cell death, defined as positive staining for Annexin V and/or PI (Q1-Q3) with increasing drug doses. The EC_50_ dose group had 54.5% cell death.

To assess cell death in 3D, we used confocal microscopy and stained for live, apoptotic, and necrotic markers (Supplementary Fig. S7d and e). OV90 cells cultured on pPerMa were treated with carboplatin at three different doses (10 μM, 50 μM, and 100 μM) for 72 h. The scaffolds were stained with CytoCalcein Violet, labelling the cytoplasm of viable cells, Apopxin Green, a phosphatidylserine (PS) sensor labelling membranes of apoptotic cells, and 7-AAD, a DNA intercalator labelling cell nuclei in membrane-permeable necrotic cells. All dyes stained OV90 cells growing on pPerMa. The fluorescent signal could be quantified by the IMARIS image analysis software using the surfaces function for live cells and the spots function for apoptotic and necrotic cells (Supplementary Fig. S7e). However, the Apopxin Green dye also stained the scaffold itself, and the staining of apoptotic cells was challenging to distinguish from background staining. For further therapy sensitivity testing, we chose to continue with only markers for live and necrotic cells.

**Fig. 7:**
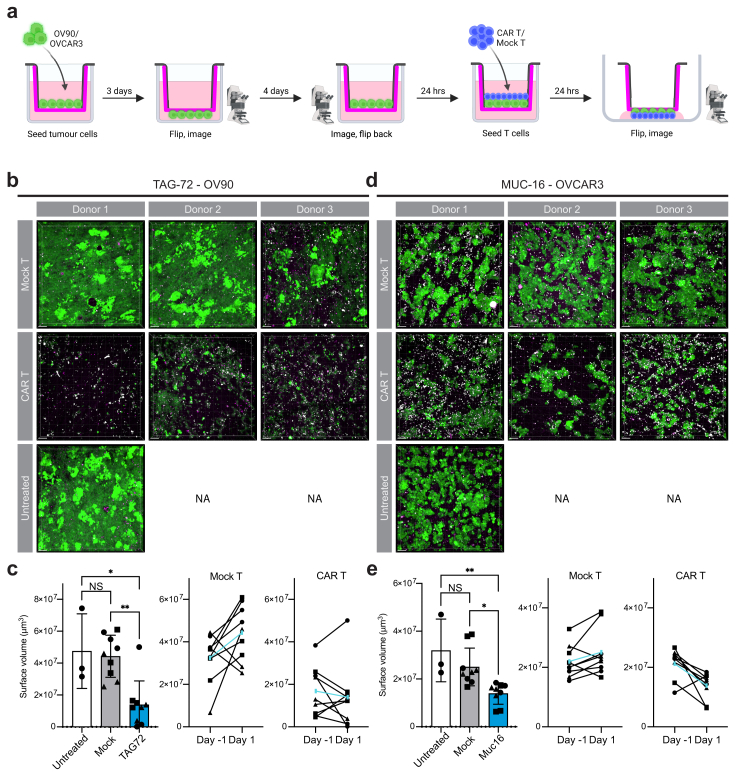
**CAR T cell sensitivity is assessed in the 3D cell cultures using confocal microscopy. a** Schematic illustration of the setup for treatment with CAR T or Mock T cells. The EOC cell lines OV90 and OVCAR3 were seeded on PerMa. OV90 cells were seeded at 25,000 cells/scaffold and OVCAR3 cells at 100,000 cells/scaffold. After three days, the scaffolds were flipped and imaged with confocal microscopy. After four more days, the scaffolds were imaged again and flipped back to the original position with the cells in the inner chamber. The next day, CAR T or Mock T cells were seeded on the scaffolds. After 24 h, the scaffolds were flipped, stained with 7-AAD, and imaged with confocal microscopy. **b**, **d** Representative confocal images of OV90 cells treated with CAR T cells targeting TAG72 or Mock T cells (b), and OVCAR3 cells treated with CAR T cells targeting Muc16 or Mock T cells (d). CAR T and Mock T cells were derived from three different donors. The experiment was performed with three replicates in each group. Magenta represents 7-AAD (ex = 561 nm, em = 575–625 nm), green represents GFP + EOC cell lines (ex = 488 nm, em = 500–550 nm), and white represents CellTraceTM Violet stained T cells (ex = 405 nm, em = 425–475 nm). The T cells that were adhered to the scaffold and/or tumour cells, did not detach when the scaffolds were flipped and can be seen in white on the confocal images. Scale bars = 100 μm. NA = not applicable. **c**, **e** Graphs showing the estimated cell volume of OV90 cells (c) and OVCAR3 cells (e) untreated and treated with Mock T cells or CAR T cells (left). Graphs showing the change in estimated cell volume from the day before treatment to 24 h after treatment for Mock T cells (middle) and CAR T cells (right). The cell volume was estimated using the surfaces function in the IMARIS image analysis software. Data represent mean ± SD, ∗p < 0.05, ∗∗p < 0.01. Different symbols in the graphs represent donor 1 (circle), donor 2 (square), and donor 3 (triangle).

To investigate the feasibility of our model system to assess sensitivity to novel cell-based therapies, we tested co-culture with CAR T cells. A CAR T cell product, directed towards tumour-associated glycoprotein 72 (TAG-72), was tested on TAG-72 positive OV90 cells. The CAR and Mock T cells were generated from PBMCs of three different healthy donors. The EOC cell lines were cultured on pPerMa for 8 days before treatment (Fig. 7a). Imaging was done on day seven to ensure growth on all scaffolds and assign them to treatment groups. On day eight, CellTrace Violet-stained T cells were seeded. After 24 h, the scaffolds were flipped, stained with 7-AAD, and imaged with confocal microscopy. The volume of live tumour cells was estimated from the GFP + signal using the surfaces function in the IMARIS image analysis software. The confocal images show the difference in live cells (green) and dead cells (magenta) in the treated and untreated groups (Fig. 7b–d). After 24 h of treatment, there was a significant difference in live cell volume between OV90 cells treated with TAG-72 targeted CAR T cells (n = 9) compared to Mock T cells (n = 9, p = 0.0015) and untreated OV90 cells (n = 3, p = 0.0111; Fig. 7c). There was also a significant difference in live cell volume between OVCAR3 cells treated with MUC-16 targeted CAR T cells (n = 9) compared to Mock T cells (n = 9, p = 0.0149) and untreated (n = 3, p = 0.0054; Fig. 7e). In summary, our 3D *in vitro* model system on pPerMa can be used to assess sensitivity to traditional and novel therapies against EOC.

## Discussion

Peritoneal carcinomatosis is frequently associated with late-stage HGSOC and is a major therapeutic challenge. In this study, we established and characterised a 3D *in vitro* model of HGSOC peritoneal metastases utilising a peritoneal dECM scaffold. Subsequently, we employed the model system to evaluate sensitivity to traditional chemotherapy and novel CAR T cell therapy.

Various advanced 3D *in vitro* models, ranging from patient-derived organoids (PDOs) to multicellular organotypics using artificial or tissue-derived hydrogels as scaffolds have been developed.^28^^,^^59^^,^^60^ These are limited in the recapitulation of the complex structure, biomolecular composition, and mechanical properties of the ovarian tumour ECM. Biological matrices made from the decellularisation of tissues have proven to maintain the complexity of the native tissue ECM and have several advantages over hydrogels derived from one or a few ECM proteins.^30^ Herbert et al. utilised a porcine SIS (pSIS) scaffold to model peritoneal EOC metastases and demonstrated the utility of their model for studying tumour cell adhesion.^32^ However, differences in the ECM of the small intestine and the peritoneum may limit the SIS model's representation of the peritoneum. Puttock et al. established a model of HGSOC omental metastases using a dECM scaffold.^33^ In a similar line of research, Sensi et al. generated a patient-derived 3D model using dECM from metastatic HGSOC tumour tissue.^34^ The omentum is phenotypically distinct from the parietal peritoneum, particularly in the immune composition.^8^^,^^10^^,^^61^^,^^62^ To establish a model that more accurately mimics the ECM of the parietal peritoneum, we developed a porcine peritoneal matrix (pPerMa). We seeded cells on both pSIS and pPerMa and noted significant differences in cellular proliferation rates between the two scaffolds (Fig. 6, Supplementary Figs. S5 and S6). Multiphoton microscopy and histological staining of major ECM components revealed only minor differences in collagen fibre arrangement, density and distribution of elastic fibres as well as mucins between pSIS and pPerMa (Fig. 3a–e). These variations are unlikely to fully account for the observed differences in cell growth on the two scaffolds. McKenzie et al. showed that higher stiffness in the environment leads to more focal adhesion formation, random migration, and migration directed by stiffness gradients (durotaxis) of tumour cells.^63^ This corroborates findings from studies reporting that tumour extracellular matrices often exhibit increased ECM rigidity.^64^^,^^65^ We did observe differences in the mechanical properties and the permeability between pSIS, pPerMa, and hPerMa; pPerMa was more rigid than both hPerMa and pSIS, while hPerMa was the least permeable. However, given the relatively minor variations in the rigidity of the pSIS, the pPerMa, and the hPerMa in our study, it is unlikely that the mechanical properties of the examined dECM scaffolds had a significant impact on the cultured cells. In contrast to common culture surfaces, such as polyethylene membranes that typically possess elastic moduli ranging from 2 GPa to 2.7 GPa, the tested biological matrices closely mimic the soft structure of the native ECM.^66^ Consequently, they are a preferable option over artificial culture substrates.

Proteomic analysis showed that the pSIS and the pPerMa preserved the diverse composition of ECM proteins of the native tissues, including collagens, glycoproteins, and proteoglycans after decellularisation (Fig. 5). The binding of ECM ligands to cell surface receptors, such as integrins, stimulates intracellular signalling pathways, facilitates cell adhesion, and regulates tumour cell migration.^67^^,^^68^ Several of the glycoproteins and proteoglycans identified among the top 10 most abundant proteins in our samples, such as lumican (LUM), decorin (DCN), fibrinogen (FG), and biglycan (BGN), are dysregulated in cancer.^68^ We also identified important regulators of post-translational modification of ECM proteins, including transglutaminase (TGM) and cathepsin (CTS) as abundant proteins, reflecting the diversity of ECM proteins in our samples.^68^ In contrast, many hydrogels consist of only one or a few different proteins. A recent study found that Matrigel®, commonly used to culture PDOs, is composed of more than 96% glycoproteins, 1% proteoglycans, and 0.4% collagen, which less faithfully replicates the characteristics of native tissue ECMs.^69^ Interestingly, we found differences in the matrisome profiles of the different dECM scaffolds (Fig. 5, Supplementary Fig. S4). First, the protein variety, or the total number of identified proteins, was comparable between the pPerMa and the hPerMa but was significantly higher in the pSIS (Supplementary Fig. S4). Second, the proportion of glycoproteins and proteoglycans was lowest in the pSIS but comparable between the pPerMa and PerMa from patients with ovarian cancer (ocPerMa) (Fig. 5). These proteomic differences could influence the cell–matrix interactions in our 3D models. Given that both the pSIS and the pPerMa aim to mimic the ECM of native tissues, differences in their structure, composition, and mechanical properties underscore the importance of carefully selecting the appropriate tissue for model establishment.

We propose a porcine-derived PerMa as a suitable model due to its similarity to the hPerMa, its availability, and its reproducibility. Principal component analysis (PCA) confirmed high similarity between biological replicates in all groups, indicating little batch-to-batch variation, which is important to ensure experimental reproducibility. The comparison of the pPerMa and the hPerMa revealed similar organisation and abundance of major ECM proteins (Fig. 3) and comparable compositions of core matrisome and matrisome-associated proteins (Fig. 5). While pPerMa and hPerMa had similar proteomic profiles, we also observed differences in the abundance of individual proteins (Fig. 5 c-f and h). Further, we recognise the potential for proteomic disparities between the pPerMa and the hPerMa that our analysis did not reveal due to the challenges of directly comparing proteins from different species. The differences in the proteomes of pPerMa and hPerMa could influence the interaction between cells and the scaffolds *in vitro*. As pointed out above, the rheological analysis uncovered differences in the viscoelastic properties of the pPerMa and the hPerMa and the permeability assay revealed a lower permeability of the hPerMa compared to pPerMa. However, the differences are smaller compared to other cell culture substrates. We confirmed the advantages of using porcine material, including the availability of tissue, the low batch-to-batch variation in the structure, mechanical properties and biomolecular composition also reported in other publications using porcine material, in addition to pigs' anatomic resemblance to humans.^37^^,^^69^, ^70^, ^71^ Overall, the pPerMa can serve as a relevant biological matrix to study peritoneal metastases. Nevertheless, there are potential limitations of our model when employing animal tissues to model human disease, as emphasised by the observed proteomic and mechanical differences between pPerMa and hPerMa. Further, static cell cultures are limited in their ability to model the physiological microenvironment of the abdominal cavity, including the impact of shear stress from ascites flow. To better replicate human biology, the human PerMa established in this study could form the basis for the development of 3D models in future studies using primary patient cells on matched ocPerMa from the same patient. The addition of a dynamic cell culture setup incorporating fluid flow would further enhance the model's representation of human disease.

We established 3D cell cultures by incorporating different cell lines in mono- and co-cultures. Cell lines offer advantages over primary cells as they are readily available and easy to use. We utilised cancer cell lines transduced to express GFP along with a fibroblast cell line transduced to express miRFP670. This enabled us to follow growth with confocal microscopy and study the interaction between the two cell types (Fig. 6, Supplementary Figs. S5 and S6). In our study, we observed that fibroblasts migrated deep into the submesothelial stroma while tumour cells remained in the more superficial layers, similar to what is seen in the native peritoneum and peritoneal metastases (Fig. 6).^8^^,^^10^ Further, in the coculture model of Caov3 cells and fibroblasts, the tumour cells formed clusters surrounded by fibroblasts in a pattern that is frequently seen *in vivo* (Fig. 6).^72^ These findings may indicate that the PerMa plays a role in guiding cell migration and organisation within tissues, which has been demonstrated for stem cells cultured on other dECM scaffolds.^37^ The pivotal ability to direct cell organisation is crucial for integrating various cell types into a multicellular culture, thereby enhancing the representation of the TME. A limitation of this study is the use of cell lines rather than primary patient cells, and the limited representation of the TME by only two cell types. Incorporating additional cell types, such as endothelial, mesothelial, and immune cells is essential for improving the fidelity of our model. Malacrida et al. successfully replicated key elements of omental metastases in a coculture model consisting of mesothelial cells, fibroblasts, adipocytes, and HGSOC cells.^73^ Utilising the PerMa as a scaffold could enable a similar reconstruction of peritoneal metastases.

Finally, we demonstrated the utility of our model for assessing sensitivity to traditional and novel cell-based therapies. Several drug screening platforms for HGSOC utilising organoid technology have emerged, demonstrating potential in replicating patient responses to therapies and suitability for high-throughput drug screening.^24^^,^^26^^,^^27^ These systems commonly use quantitative assays that measure viability by detecting cellular metabolism via, for example, adenosine triphosphate (ATP). Our drug screening method differs technically from these platforms in several aspects, providing significant advantages. The custom-designed culture plate insert (referred to as a “crown”, Fig. 2) enabled us to set up the scaffolds in a Transwell®-like system with culture medium on both sides. The crown was adapted for confocal imaging. It can be downscaled to fit smaller culture plate wells, making it suitable for experiments with limited material availability. Our crown setup additionally allows for the simultaneous setup of migration and invasion assays, along with advanced imaging capabilities. With this configuration, we tracked the growth of the 3D cell cultures using confocal microscopy, both in standard cell culture conditions and during the therapy sensitivity assays and quantified live cells to assess the efficacy of chemotherapy and CAR T cell therapy (Figs. 6 and 7, Supplementary Figs. S5–S7). While frequently used bioluminescence assays are faster and easier to quantify, they do not provide the ability to examine cellular interaction, which is particularly advantageous for investigating novel cell-based immunotherapies. Our model is not a high-throughput screening platform but a highly valuable model to validate findings from other *in vitro* screening platforms to further explore how the interactions between cells and matrices could impact the therapeutic response of drugs and cell-based therapies.

In conclusion, we successfully established a 3D *in vitro* model of HGSOC peritoneal metastases using the pPerMa as a biological scaffold. The characterisation demonstrated that the pPerMa closely recapitulates the ECM of the native peritoneum, making it a relevant platform for studying the unique TME of peritoneal metastases. Cell lines cultured on the PerMa show similar growth patterns to HGSOC *in vivo*. We demonstrated the utility of our model system for assessing sensitivity to traditional chemotherapy and novel cell-based immunotherapies. Although pPerMa shares several key features with human-derived PerMa and can serve as a useful model for studying HGSOC peritoneal metastases, we also identified distinct differences that highlight the need for further research focused on developing and optimising human-derived PerMa scaffolds for *in vitro* culture. In future studies, we aim to establish 3D cell cultures using primary tumour samples from HGSOC patients cultured on patient-matched ocPerMa scaffolds. Overall, our model can serve as a valuable tool for the development of effective therapies for peritoneal carcinomatosis in HGSOC.

## Contributors

C.H.G. Conceptualisation, investigation, methodology, visualisation, writing—original draft, writing—review and editing, access and verification of the underlying data.

K.K. Conceptualisation, investigation, methodology, supervision, writing—review and editing, access and verification of the underlying data.

A.L. Methodology, investigation, supervision.

G.R.G.P Methodology, investigation, supervision.

E.R. Investigation, methodology.

G.N.S Investigation.

C.L. Methodology.

R.E. Investigation, methodology.

H.N.D. Investigation, methodology.

C.B. Investigation, methodology, visualisation, writing—original draft.

O.G. Investigation, methodology, software.

C.F. Investigation, methodology.

M.S. Investigation, methodology.

T.A.N. Funding acquisition, methodology, resources.

K.L. Funding acquisition, methodology, software.

B.D. Investigation, methodology, resources.

S.W. Methodology, resources.

P.G. Methodology.

D.E.C. Methodology.

S.K. Investigation, methodology, resources.

L.B. Conceptualisation, funding acquisition, project administration, resources, supervision, writing—original draft, writing—review and editing.

E.M. Conceptualisation, funding acquisition, project administration, resources, supervision, writing—review and editing.

All authors read and approved the final version of the manuscript.

## Data sharing statement

The mass spectrometry proteomics data have been deposited to the ProteomeXchange Consortium via the PRIDE partner repository with the dataset identifier PXD049045.^41^ Other data will be made available on request after publication.

## Declaration of interests

L.B. has received honoraria for lectures from GlaxoSmithKline, Merck Sharp, and Dohme and has received a research grant from AstraZeneca for a researcher-initiated trial. L.B. has had leadership roles in Onkologisk Forum between 2018 and 2022 and in the Nordic Society of Gynaecological Oncology (NSGO) and NSGO-Clinical Trial Unit since 2021. She is a faculty member of the Gynaecological Cancers Group European Society of Medical Oncology (ESMO) external expert in gynaecologic oncology for the Norwegian National Expert Panel for Persons with Serious Life-Shortening Diseases and Member of the national specialist group in oncology Sykehusinnkjøp HF. She is also a board member for KinN Therapeutics AS–a biopharmaceutical company focused on tailored preclinical oncology services for the development of novel anticancer compounds and Oslo Cancer Cluster, an innovation environment in Oslo. E.M.C. is co-founder, shareholder and board member of KinN Therapeutics AS. B.D. is a consultant and speaker for MSD, AZ and GSK. C.B. has received Bridging Funding, IZKF, University Hospital Würzburg and Add-On-Fellowship, Joachim Herz Stiftung.

## Appendix B Supplementary data

Supplementary File 3

Mycoplasma testing

STR profiling compiled

ICLAC_Cell-Line-Checklist

Supplementary Figures and tables Caption
