# Supplementary material for: A 3D ovarian cancer metastasis model using a decellularised peritoneal matrix to study therapy response
Source: eBioMedicine. 2026 Feb 2;124:106135. doi: 10.1016/j.ebiom.2026.106135 (PMC12887378; doi:10.1016/j.ebiom.2026.106135)

Supplementary fig. 1

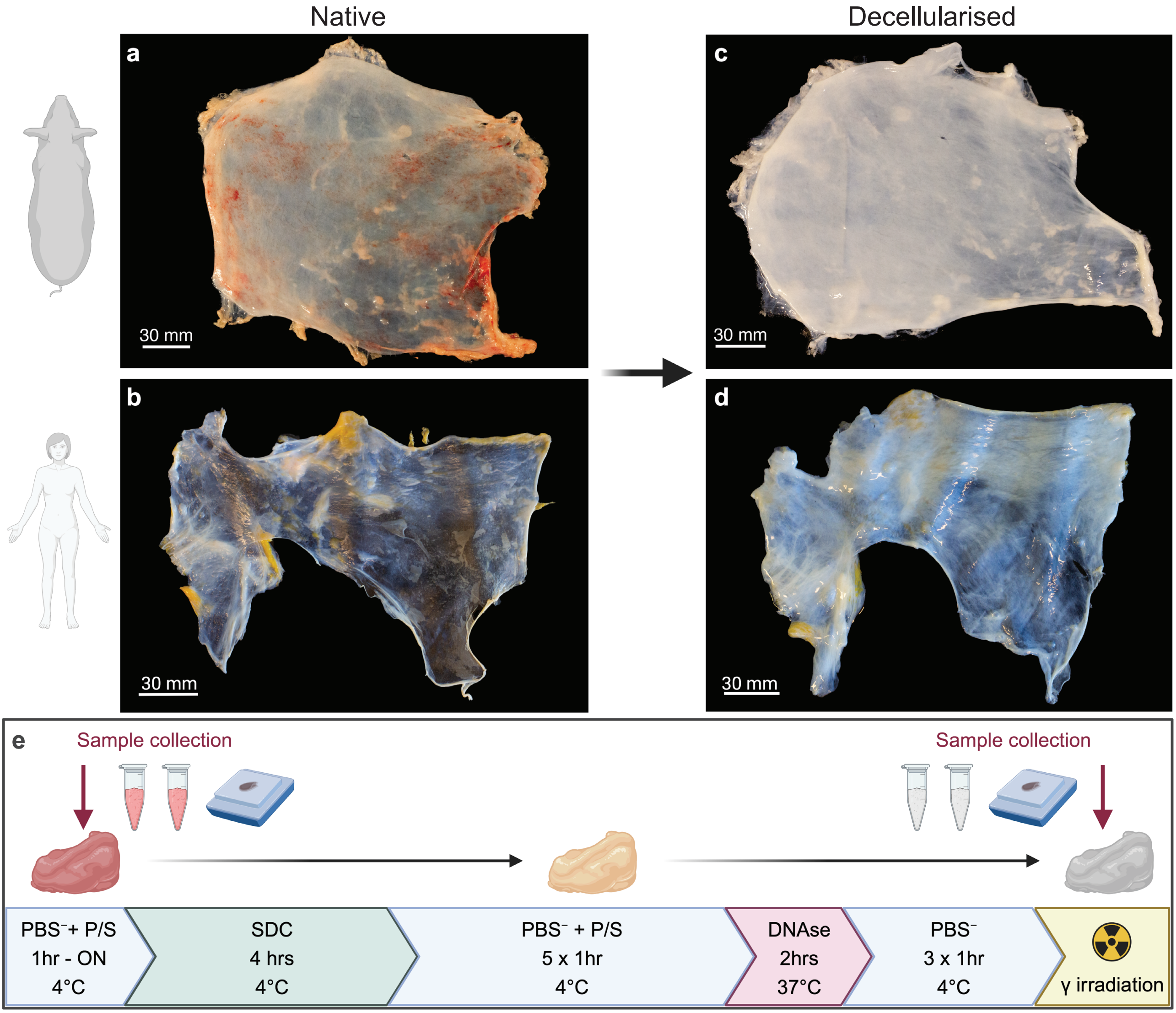

Supplementary fig. 2

a Crown

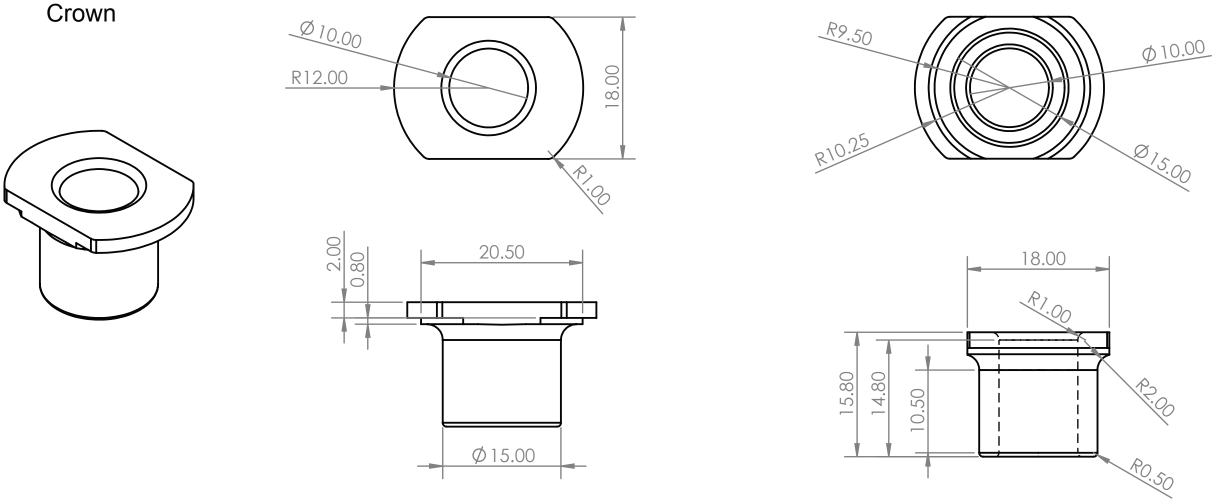

b Attachment clip

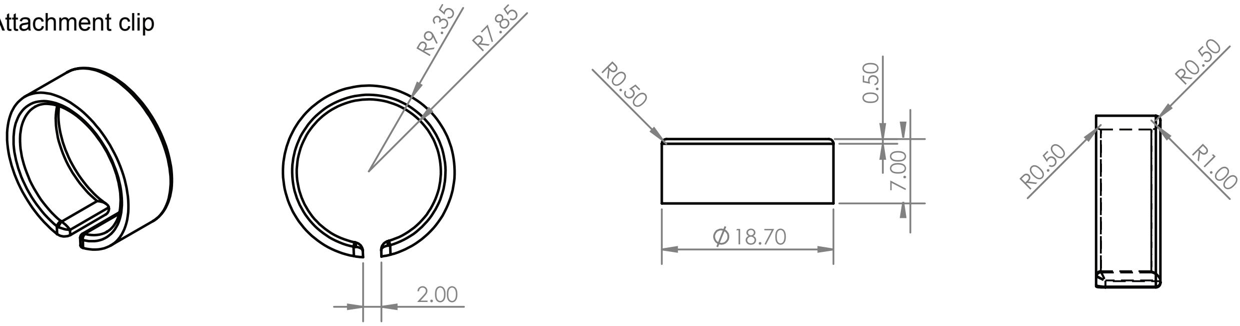

c Staining ring

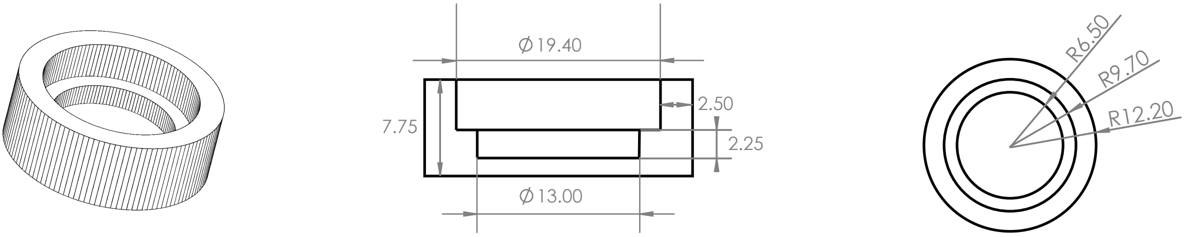

Supplementary fig. 3

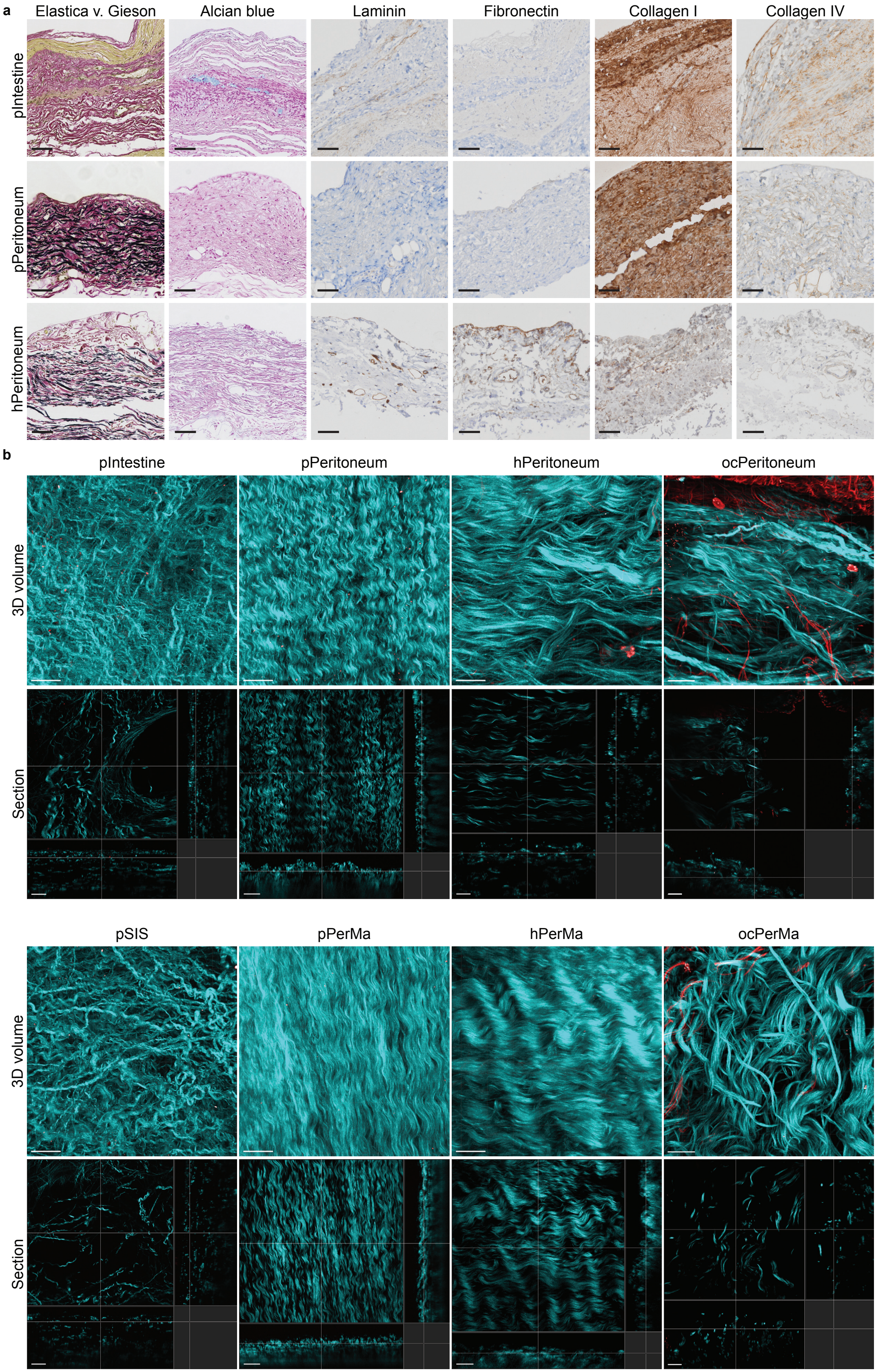

Supplementary fig. 4

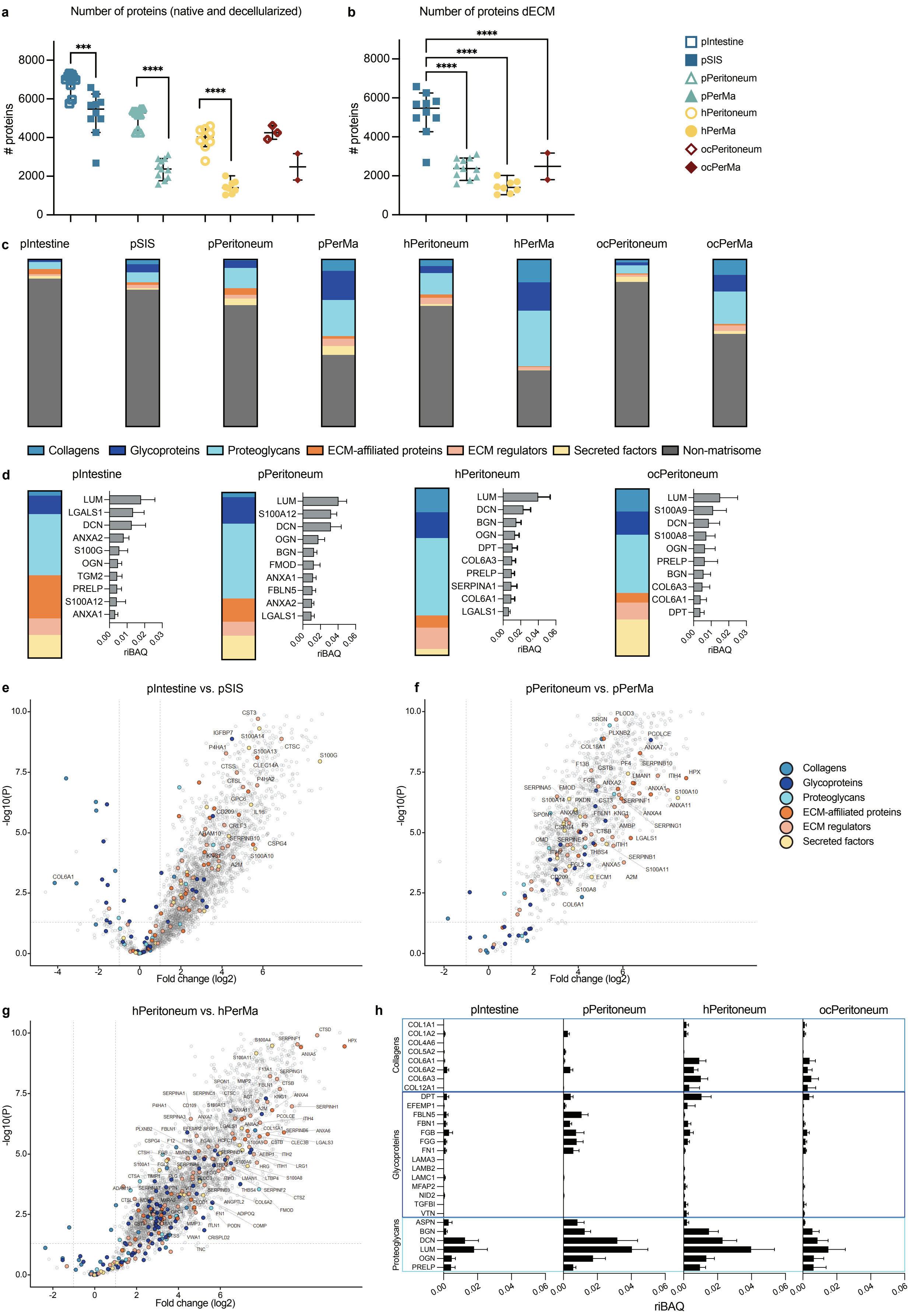

Supplementary fig. 5

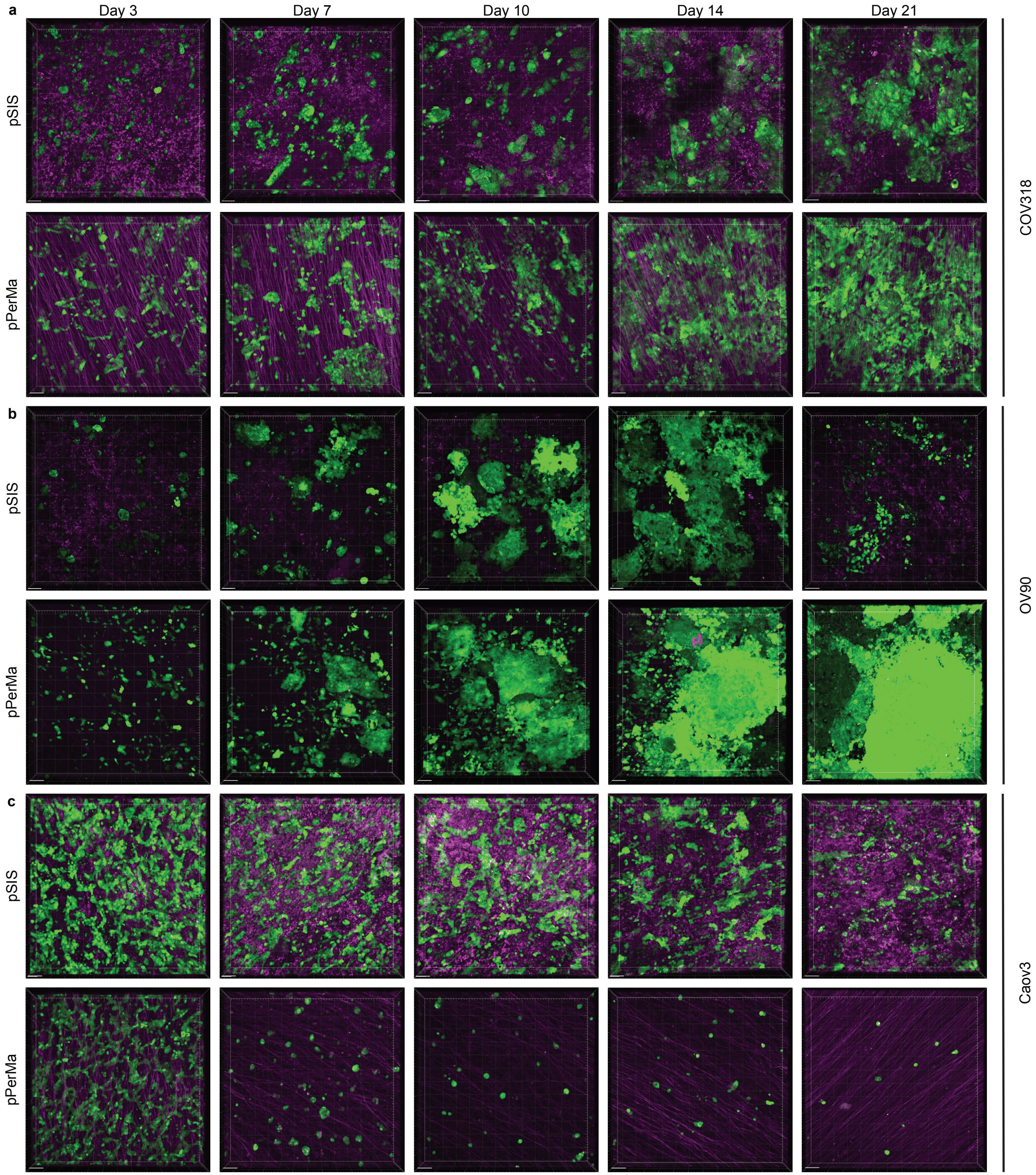

Supplementary fig. 6

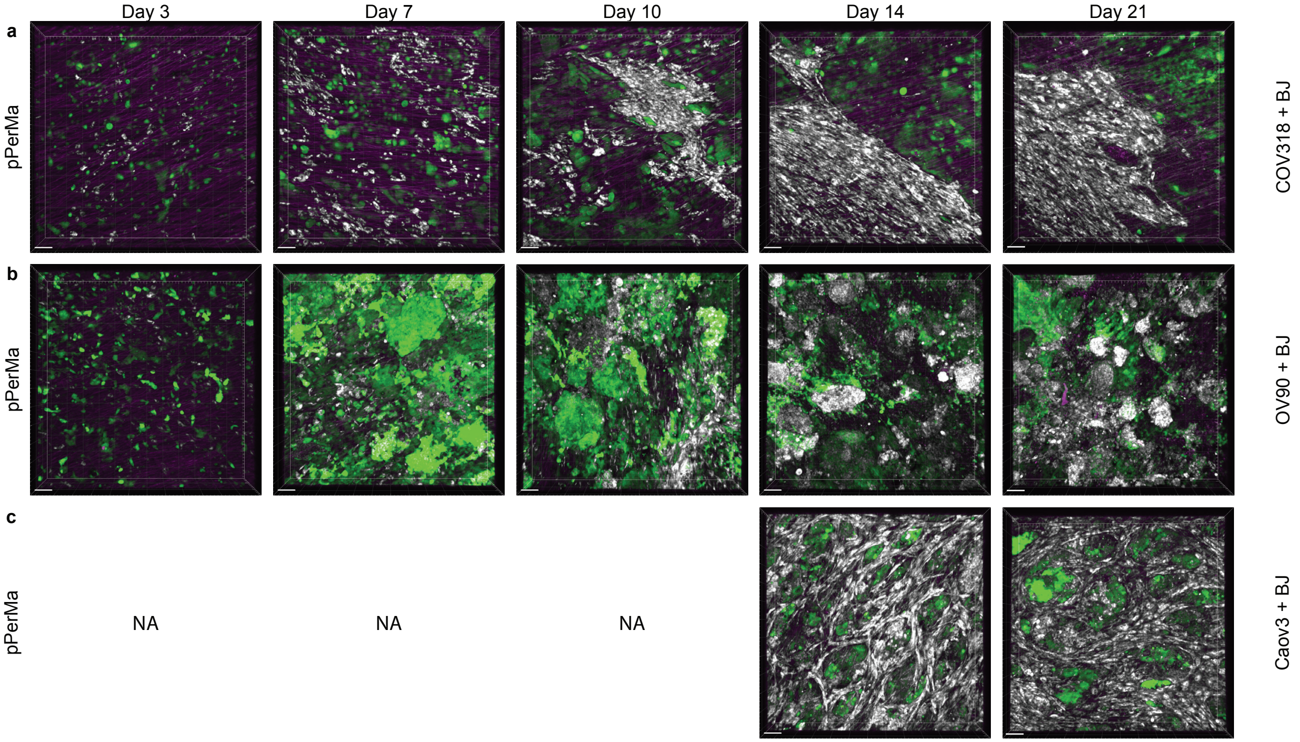

Supplementary fig. 7

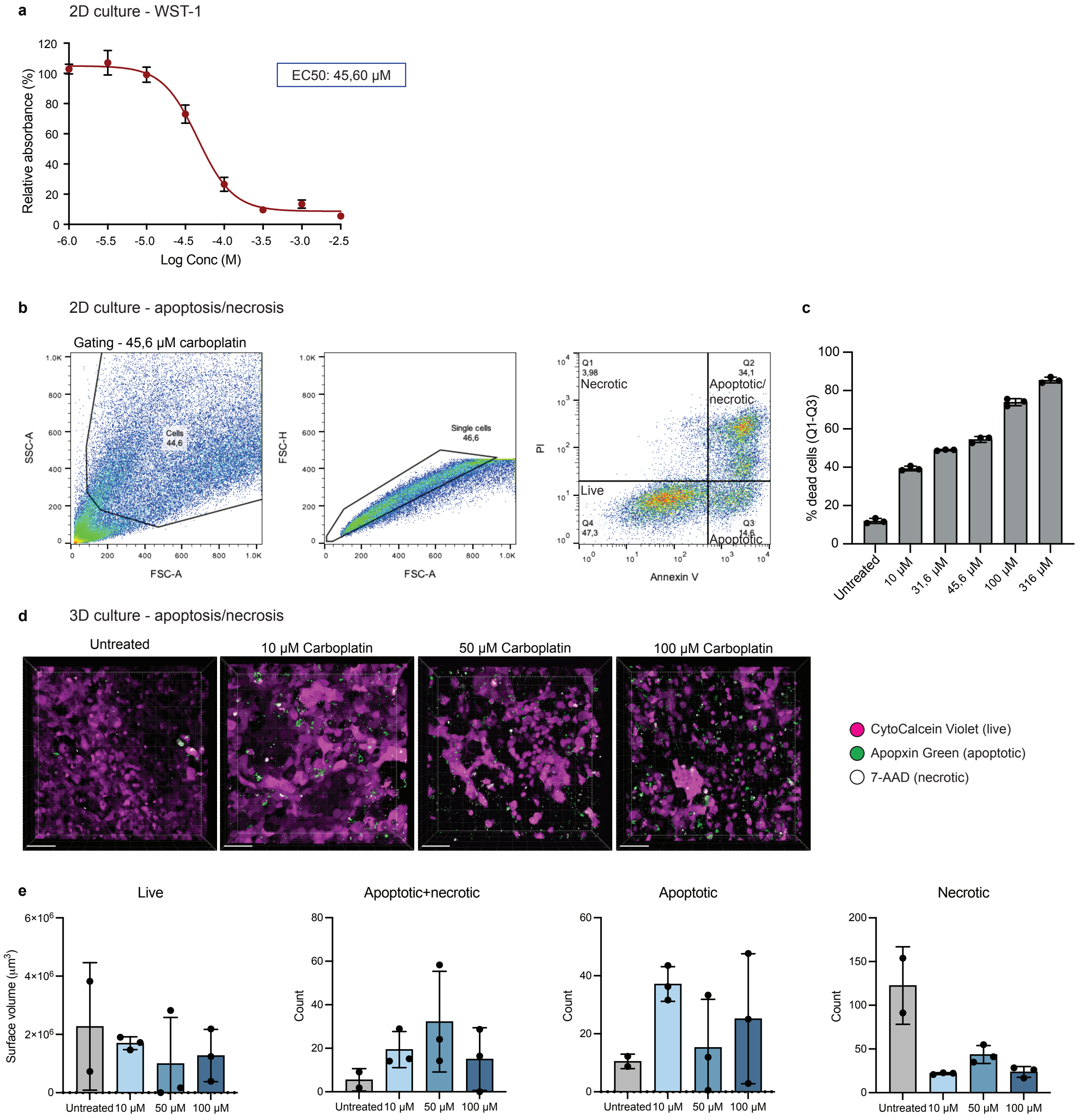

Supplement: Supplementary Figures [file mmc1.pdf]
