# Supplementary material for: A 3D ovarian cancer metastasis model using a decellularised peritoneal matrix to study therapy response
Source: eBioMedicine. 2026 Feb 2;124:106135. doi: 10.1016/j.ebiom.2026.106135 (PMC12887378; doi:10.1016/j.ebiom.2026.106135)
Supplement: Supplementary Tables [file mmc2.docx]

**Supplementary Table 1.**

| Antibody | Manufacturer | Cat. # | RRID | Clone | Host | Dilution | Antigen retrieval | Positive control |
| --- | --- | --- | --- | --- | --- | --- | --- | --- |
| Collagen I | Proteintech (Nordic Biosite) | 67288-1-Ig-20 | AB_2882554 | 1E9A7 | Mouse | 1:3000 | HpH (pH 9) | Normal human colon |
| Collagen IV | Invitrogen (Thermo Fisher) | PA1-28534 | AB_1956972 | Polyclonal | Rabbit | 1:400 | LpH (pH 6) | Normal human colon |
| EpCAM | Abcam | Ab20160 | AB_445379 | AUA1 | Mouse | 1:200 | LpH (pH 6) | Human breast carcinoma |
| Fibronectin | NeoBiotechnologies | 2335-MSM2-P0 |  | TV-1 | Mouse | 1:100 | HpH (pH 9) | Human kidney |
| Laminin | Sigma Bio Sciences | L-8271 | AB_477162 | LAM-89 | Mouse | 1:800 | LpH (pH 6) | Human kidney |

**Supplementary Table 2.**

| Patient | Age | EOC subtype | Stage | Chemonaïve |
| --- | --- | --- | --- | --- |
| hPerMa1 | 90 | NA | NA | NA |
| hPerMa2 | 82 | NA | NA | NA |
| hPerMa3 | 72 | NA | NA | NA |
| hPerMa4 | 66 | NA | NA | NA |
| hPerMa5 | 55 | NA | NA | NA |
| hPerMa6 | 71 | NA | NA | NA |
| hPerMa7 | 74 | NA | NA | NA |
| hPerMa8 | 57 | NA | NA | NA |
| hPerMa9 | 92 | NA | NA | NA |
| ocPerMa1 | 63 | HGSOC | IIIc | Yes |
| ocPerMa2 | 61 | HGSOC | IVb | No |
| ocPerMa3 | 62 | HGSOC | IVa | Yes |
