# Supplementary material for: A 3D ovarian cancer metastasis model using a decellularised peritoneal matrix to study therapy response
Source: eBioMedicine. 2026 Feb 2;124:106135. doi: 10.1016/j.ebiom.2026.106135 (PMC12887378; doi:10.1016/j.ebiom.2026.106135)
Supplement: Supplementary Figures and tables Caption [file mmc9.docx]

**CAPTION FOR SUPPLEMENTARY MATERIAL**

**Supplementary figure 1.** Photographs showing the native porcine (**a**) and human (**b**) peritoneum and the decellularized porcine (**c**) and human (**d**) PerMa. Scale bars 30 mm. **e** Schematic illustration of the protocol for decellularization of human and porcine peritoneum. Before decellularization, specimens were collected for formalin fixation and paraffin embedding (FFPE), and storage at -80 °C. Samples were washed in PBS with penicillin/streptomycin (P/S) for a minimum of 1 hour, maximum overnight (ON). Samples were incubated in sodium deoxycholate (SDC) for 4 hours at 4°C, followed by washing in PBS with P/S for a minimum of 5 x 1 hour at 4°C. Then, samples were incubated in DNAse solution for 2 hours at 37°C, followed by 3 x 1 hour washing in PBS. After decellularization, specimens were collected for FFPE and storage at -80°C. The scaffolds were then irradiated with gamma irradiation for sterilization.

**Supplementary figure 2.** Sketches showing the dimensions of the cell culture crown (a), attachment clip (b), and staining ring (c).

**Supplementary figure 3. a** Representative images of Elastica v. Gieson and Alcian blue staining and immunohistochemical staining of laminin, fibronectin, collagen I, and collagen IV of pIntestine, pPeritoneum, and hPeritoneum. Scale bars 50 µm. **b** Representative second harmonic generation (SHG; cyan)/two-photon excitation fluorescence (TPEF; red)) microscopy images of pIntestine, pPeritoneum, hPeritoneum, and ocPeritoneum (top panel) and pSIS, pPerMa, hPerMa, and ocPerMa (bottom panel). 3D renderings (top) and single optical sections (bottom). Scale bars = 50 µm. The 3D renderings of the pSIS, pPerMa, hPerMa, and ocPerMa are the same as are displayed in Fig. 3b.

**Supplementary figure 4.** Proteomic analysis by liquid-chromatography mass spectrometry (LC-MS/MS). **a** Number of identified proteins in each group. Data represent mean ± SD. *** P < 0.001, **** P < 0.0001, unpaired two-tailed t-test performed for each pair of native/decellularized tissue. **b** Number of identified proteins in the dECM scaffolds. Data represent mean ± SD. **** P < 0.0001, one-way ANOVA with Tukey’s multiple comparison test. **c** The composition of matrisome and non-matrisome proteins in all groups. **d** The composition of total matrisome proteins and the ten most abundant matrisome proteins in pIntestine (n = 10), pPeritoneum (n = 11), hPeritoneum (n = 9), and ocPeritoneum (n = 3). The ratios of the top ten proteins are given as iBAQ intensity relative to the total iBAQ intensity (riBAQ) for each sample. Data represent mean ± SD. **e-g** Volcano plot of protein intensities in pIntestine (n = 10) vs. pSIS (n = 10; d), pPeritoneum (n = 11) vs. pPerMa (n = 11; e), and hPeritoneum (n = 9) vs. hPerMa (n = 8; f). Matrisome proteins with at least a 3-fold change (log2) are labeled. **h** Profile bar charts for pIntestine (n = 10), pPeritoneum (n = 11), hPeritoneum (n = 9), and ocPeritoneum (n = 3) of selected core matrisome proteins (n = 28). The proteins are sorted into collagens (top box), glycoproteins (middle box), and proteoglycans (bottom box). The ratios are given as relative iBAQ intensities. Data represent mean ± SD.

**Supplementary figure 5.** Representative confocal images of COV318 (**a**), OV90 (**b**), and Caov3 (**c**) on pSIS (top) and pPerMa (bottom) on days 3-21. COV318 and OV90 cells were seeded in a number of 25,000 cells/scaffold, while Caov3 cells were seeded at 400,000 cells/scaffold. The confocal images are 3D renderings of z-stacks, visualized in the x/y plane. Magenta represents tissue autofluorescence (ex = 561 nm, em = 575-625 nm) and green represents GFP+ EOC cell lines (ex = 488 nm, em = 500-550 nm). Scale bars = 100 µm.

**Supplementary figure 6.** Representative confocal images of BJ fibroblasts in coculture with COV318 (**a**), OV90 (**b**), and Caov3 (**c**) on days 3-21. COV318 and OV90 cells were seeded in a number of 25,000 cells/scaffold and Caov3 cells at 200,000 cells/scaffold. For cocultures, BJ fibroblasts were seeded in a number of 12,500 cells/scaffold (COV318), 6,250 cells/scaffold (OV90), and 25,000 cells/scaffold (Caov3). The confocal images are 3D renderings of z-stacks, visualized in the x/y plane. Magenta represents tissue autofluorescence (ex = 561 nm, em = 575-625 nm), green represents GFP+ EOC cell lines (ex = 488 nm, em = 500-550 nm), and white represents miRFP670+ BJ fibroblasts (ex = 637 nm, em = 663-738 nm). Scale bars = 100 µm. NA = not applicable (only imaged on days 14 and 21).

**Supplementary figure 7.** Carboplatin sensitivity for OV90 cells. **a** Dose-response curve for OV90 cells in 2D culture, treated with different doses of carboplatin. Y axis represents the absorbance of formazan in treated cells relative to control (%). X axis represents doses of carboplatin in moles/liter (M). The EC50 drug dose was determined to be 45.60 µM. **b,c** Response to carboplatin for OV90 cells in 2D culture, evaluated with Annexin V/PI staining and flow cytometry. **b** Example gating shown for one sample treated with 45.6 µM carboplatin. **c** Graph showing the percentage of dead cells for the different doses of carboplatin and untreated, calculated as the sum of Q1-Q3. Data represent mean ± SD. **d** Representative confocal images of OV90 cells cultured on pPerMa and treated with carboplatin at different doses (10 µM, 50 µM, and 100 µM). The confocal images are 3D renderings of z-stacks, visualized in the x/y plane. Magenta represents live cells stained with CytoCalcein Violet (ex = 405 nm, em = 425-475 nm), green represents apoptotic cells stained with Apopxin Green (ex = 488 nm, em = 500-550 nm), and white represents necrotic cells stained with 7-AAD (ex = 637 nm, em = 663-738 nm). **e** Graphs showing the surface volume (left graph) and cell count (three right graphs) of the markers for live, apoptotic, and necrotic cells for scaffolds treated with increasing doses of Carboplatin. The second graph indicates cells that stained double positive for the apoptotic and the necrotic markers, while the two graphs two the right indicate cells that were positive for only one marker. Data represent mean ± SD.

**Supplementary Table 1:** List of antibodies used for immunohistochemistry staining. HpH = high pH, LpH = low pH.

**Supplementary Table 2:** Human cohort demographics. NA = not applicable.

**Supplementary File 1:** STL file for 3D printing of crown.

**Supplementary File 2:** STL file for 3D printing of attachment clip.

**Supplementary File 3:** STL file for 3D printing of staining ring.
